# Multi-omics analysis-based insights into the microbial community composition and flavor development potentiality of different varieties of sorghum (*Sorghum bicolor* L. Moench) fermented into Sesame flavor *Baijiu*

**DOI:** 10.1016/j.crmicr.2026.100606

**Published:** 2026-05-15

**Authors:** Meina Jiang, Jiaxin Tang, Angui Lai, Wei Sun, Xue Liu, Zhichao Shang, Zuzhong Zheng, Xiaoxue Chen, Yansong Xue, Bei-Zhong Han

**Affiliations:** a China Agricultural University-Sichuan Advanced Agricultural & Industrial Institute, Chengdu, Sichuan, 611430, China; b Key Laboratory of Functional Dairy, Co-constructed by Ministry of Education and Beijing Government, College of Food Science and Nutritional Engineering, China Agricultural University, Beijing, 100083, China; c Shandong Jingzhi Liquor Co., Ltd., Anqiu, 262119, China; d Yunnan Chishuiyuan Liquor Industry Co., Ltd., Zhaotong, 657229, China; e Key Laboratory of Jiuqu Quality and Engineering of China National Light Industry, China Agricultural University, Beijing, 100083, China

**Keywords:** Sorghum varieties, Microbial communities, Sesame-flavor Baijiu, Volatile compounds

## Abstract

Sorghum is a key raw ingredient in the fermentation of Sesame-flavor *Baijiu* (SFB). However, studies on the microbial and metabolic dynamics of sorghum varieties on SFB fermentation is limited. In this study, microbial and flavor differences during SFB fermentation of three sorghum varieties (*Hongyingzi, Hei’e*, and *Shandong*) were analyzed and compared. The physicochemical factors of sorghums influencing bacterial communities were greater than those affecting fungal communities. This is consistent with the finding that bacteria play a dominant role in shaping the differences in microbial community structures during fermentation with different sorghum varieties. In addition, the microbial interactions and their relationship with volatile compounds varied across different sorghum varieties. Meanwhile, the physicochemical factors that govern microbial succession also differed among these sorghum varieties. These variations collectively contribute to the observed differences in fermentation outcomes and product quality. These findings offer insights into the role of raw materials in solid-state fermentation. Specifically, they highlight how microbial dynamics and physicochemical conditions influence the consistency and quality of SFB production.

## Highlights

• Sesame-flavor *Baijiu* fermentation was performed using different sorghum varieties.
• Microbial interactions affected volatile formation differently in sorghum varieties.
• Cultivar-specific physicochemical traits modulate microbial consortia structure.
• Sorghum selection can format flavor paradigm and standardize *Baijiu* quality.

## Graphical abstract

**Figure:**
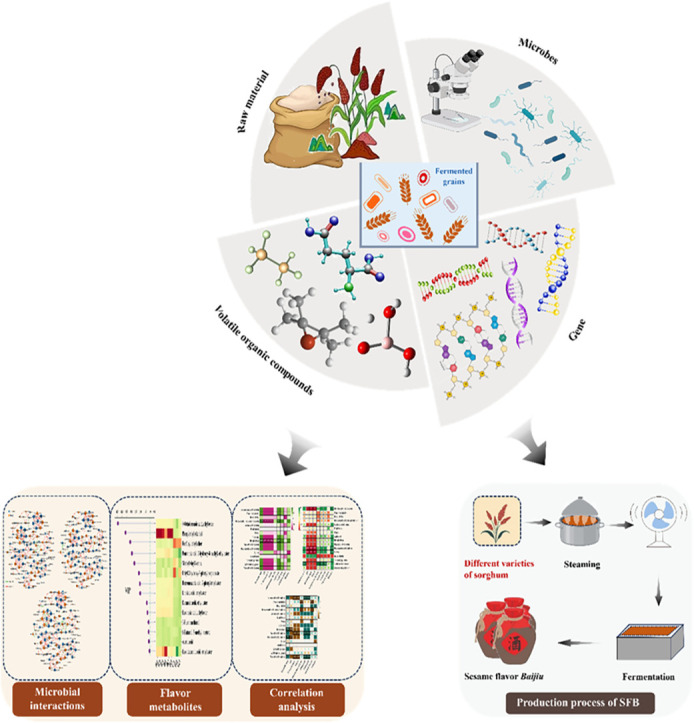

## 1 Introduction

*Baijiu* is one of the oldest distilled spirits in the world, with a rich history deeply embedded in Chinese culture and tradition (Wang et al., 2021a) . With its distinctive flavor profile, *Baijiu* has been ranked as one of the top seven distilled spirits worldwide (Cai et al., 2022). It is made from a variety of cereal grains such as sorghum, barley, wheat, rice and corn (Chen et al., 2023), with ethanol and water accounting for about 98% of its composition. The key to determine the quality of *Baijiu* is the trace flavor compounds that account for only 2% (Sun et al., 2021). Among them, Sesame-flavor *Baijiu* (SFB) has become one of the most popular aroma types of Chinese *Baijiu*, known for its rich, roasted sesame seed-like fragrance and smooth and mellow taste (Sha et al., 2017; Shen et al., 2020). The quality of *Baijiu* is predominantly defined by its flavor profile, which is shaped by both the intrinsic properties of the raw materials and the metabolic activities of microorganisms during fermentation (Ren et al., 2025; Wang et al., 2021b). While the functional dynamics of microbiota during fermentation are known to be strongly influenced by raw materials, the specific mechanisms linking physicochemical properties to microbial activity remain poorly understood.

The main raw material for SFB fermentation is sorghum grain, the quality of which is characterized by traits such as amylose content, protein and tannin levels, which are significantly influenced by the interaction of sorghum varieties and cultivation environment (Markos et al., 2020). Protein and tannin content vary widely among sorghum varieties and play a key role in determining the fermentation properties of sorghum (Andiku et al., 2021). Previous study (Wu et al., 2017) demonstrated that using sorghums with different starch composition (covering two glutinous and four japonica varieties) as fermentation substrates resulted in significant differences in *Baijiu* flavor, even when inoculated with the same functional yeast. Previous research on proteins from sorghum have found that globulins and albumins significantly elevated the number of microbial populations closely related to amino acid production (Wang et al., 2021c). However, the sorghum varietal traits govern microbial community assembly and orchestrate flavor compound biosynthesis remain unresolved, limiting predictive control over fermentation outcomes. Industry-standard practices between the two sorghum types. However, in the actual production of sesame-flavor *Baijiu*, glutinous sorghum is predominantly used. Therefore, existing research has overlooked the subtle variations among different varieties within the glutinous sorghum subgroup.

Therefore, this work is to assess the effect of different sorghum varieties (three distinct glutinous sorghum varieties) on the microbial community that shape the flavor profiles during SFB fermentation. This study will provide a scientific basis for the strategic selection of raw materials, which is essential for standardizing and enhancing the quality of *Baijiu* processing.

## 2 Materials and methods

### 2.1 Experimental design and sample collection

The solid-state fermentation experiments in this study were conducted at a traditional *Baijiu* distillery in Weifang, Shandong Province, to reflect industry-standard practices and ensure ecological relevance to real-world fermentation processes. Specifically, the mixture was first subjected to stacking fermentation. After a layer of white mycelial mat fully covered the surface of the fermented grain, it was transferred to pits for subsequent fermentation. High-temperature *Daqu* was inoculated at an amount of 10% as the starter culture. The maximum temperature reached 55 °C throughout the fermentation process. Three glutinous sorghum varieties (*Hongyingzi* sorghum, *Hei’e* sorghum, *Shandong* sorghum), were selected for the fermentation experiment. Sorghum information can be found in Table 1. Prior to initiating the *Baijiu* fermentation process, three sorghum samples were randomly collected from the storage room. Specifically, 10 kg of sorghum was sampled from each of the four diagonal positions and the upper, middle, and lower layers at the center of the grain pile. The collected samples were thoroughly mixed and then reduced to a 1 kg composite sample using the quartering method. Of this composite sample, 250 g was sealed in a glass container for reserve and re-examination, while the remaining portion was designated as the analytical sample. For the fermentation experiment, each sorghum variety was processed in triplicate pits. Three varieties of sorghum were fermented in nine pits that had been seasoned for ten years, and the fermentation experiment began on June 12, 2023 and lasted for 113 days. On days 0, 15, 50, and 113 of the fermentation process, we collected samples of the fermented grains from the four corners and the center of the middle sections of the pits (five time points in total) using a specialized sampler. Prior to each sampling, the sampler was immersed in 75% alcohol for 30 min to ensure its thorough cleaning and disinfection. The five samples (100 g per sample) collected from the same pit by the five-point method were mixed well and marked as one sample (total mass: 500 g) at the end of each sampling and subsequently transferred to sterile sampling bags. A total of 36 fermented grain samples and 9 pre-fermentation sorghum samples were collected in this study.

**Table 1:** The information of three cultivars of sorghum.

Sorghum ID	Sorghum variety	Geographical origin	Collection date	Colour
R	Hongyingzi	Guizhou province, China	2023	Red
B	Heie	Shandong province, China	2023	Red
G	Shandong	Shandong province, China	2023	Yellow

R denotes *Hongyingzi* sorghum. B denotes *Hei’e* sorghum. G denotes *Shandong* sorghum.

### 2.2 Physicochemical analysis

To understand the fermentation processes of three varieties of sorghum, we analyzed nine fermentation parameters to figure out features of fermentation stages. The Moisture of sorghum and fermented grains samples was determined using the gravimetric method, by drying 5.0 g samples at 105 °C for 3 h (Pang et al., 2020). Sorghum powder (3.0 g) was homogenized with 25 mL of distilled water and stirred at 160 rpm to prepare slurries for analyzing the thermal characteristics of starch granule using an RVA4800 instrument (Boton Ruihua, Beijing, China). Four grams of fermented grains were homogenized in 20 mL of distilled water, and the pH of the homogenate was measured using a digital pH meter (PHS-920, Youmu, Shanghai, China). Sorghum samples were homogenized with ethanol and heated at 80 °C for 30 min and the sediment were collected for quantification of total starch, amylose and amylopectin. The starch content was measured using enzymolysis kits (G0551W, Suzhou Grace Bio-technology Co., LTD, Suzhou, China), and the enzymes required for the assay were supplied by the same manufacturer. Sorghum tannins were quantified using methods described by national standards of China (China, 2008). Briefly, tannin was quantified at 525 nm after extraction with a dimethylacetamide solution, followed by reaction with ferric ammonium citrate and ammonium hydroxide to develop a measurable chromogenic complex. The reduction degree of sugars in fermented grains was determined via the 3,5-dinitrosalicylic acid (DNS) colorimetric method at a wavelength of 540 nm (Miller, 1959), and the standard curve was constructed using glucose. Ethanol content was read directly using an alcohol meter, and acetic acid and lactic acid contents were analyzed according to the method of Kang et al. (2022b). The sorghum protein was detected by Kjeldahl Method according to the national professional standard methods (China, 2016). The protease activity was determined using the Folin–Ciocalteu’s phenol reagent method. In particular, lactic acid buffer was used for the crude extraction of acid protease activity (Shu et al., 2020).

### 2.3 Volatile compound analysis

Headspace solid-phase microextraction combined with 8890–7000D gas chromatography-mass spectrometry (HS-SPME-GC–MS) (Agilent, USA) was used to assess the flavor compounds in fermented grains and *Baijiu*. Volatile components present in the samples were collected using 50/30 μm DVB/CAR/PDMS fibers (Supelco, Merck, USA). Prior to extraction, the samples were equilibrated in a temperature-controlled device at 40 °C for 30 min. Subsequently, the extraction process was carried out at 60 °C for 30 min. After extraction, the fiber, which also contained 4-methyl-2-pentanol serving as an internal standard, was introduced into the GC inlet and maintained at 250 °C for 8 min. Prior to analysis, the samples were processed on HP-FFAP columns (J&W Scientific in California, USA). The operating parameters of the GC–MS were determined according to methods that previously reported (Kang et al., 2022a). Volatile compounds detected in the samples were identified by comparing the chromatographic data with the NIST 20 chromatographic database, and the relative amounts were determined by peak area normalization.

### 2.4 Microbial community composition analysis

A 5 g fermented grains sample was weighed and genomic DNA was extracted using Omega Soil DNA Kit (Omega Biotek, Norcross, GA, USA). For DNA extraction control, a blank control with sterile double-distilled water instead of sample matrix was processed in parallel to monitor cross-contamination during the extraction procedure. Primer 799F/1193R was used to amplify the V5-V7 variable region of the 16S rRNA bacterial gene, and primer ITS1(b) was used to amplify the ITS region of the fungal gene. Specific primer information is shown in Table S2.

The PCR reaction was performed using the following procedure: pre-denaturation at 98 °C for 2 min, 35 cycles of 30 s each at 98 °C, annealing at 55 °C for 30 s, extension at 72 °C for 90 s, and a final extension at 72 °C for 5 min. The PCR reaction was performed in triplicate: 25 μL of reaction buffer containing 5 μL of reaction buffer, 5 μL of 5×GC buffer, 2 μL of dNTP (2.5 mM), 1 μL of each primer (10 μM), 2 μL of template DNA, 0.25 μL of DNA polymerase, and 8.75 μL of double-distilled water. For PCR amplification control, a no-template control (NTC) with sterile double-distilled water replacing template DNA was included in each PCR run to detect possible contamination from reagents or labware. The resulting PCR products were electrophoresed and tested by 1% agarose gel electrophoresis, and then subjected to double-end sequencing on the Illumina NovaSeq PE250 platform. Demultiplexing and quality assurance filtering were carried out using DADA 2 software. Following this, FLASH software, version 1.2.11, was used to combine the paired-end sequencing data. After discarding chimeric sequences, the remaining high-quality sequences were categorized into operational taxonomic units (OTUs) with Vsearch, adopting a 97% sequence similarity threshold. Then, representative sequences from these OTUs were matched against entries in the Silva database and the Unite fungal taxonomy database, and species annotation was performed using the classify-sklearn algorithm embedded in QIIME2.

### 2.5 Statistical analysis

To assess the dynamic changes in microbial communities during the fermentation process of different sorghum varieties, we used α-diversity as an evaluation metric. Specifically, we employed four parameters: the Shannon index and Chao 1 index, which comprehensively reflect species richness and evenness, with higher values indicating greater diversity. The Simpson index emphasizes the contribution of dominant species. Observed_species directly counts the number of OTUs (operational taxonomic units) detected in the sample, reflecting species richness. In order to determine the significance of physicochemical properties, one-way analysis of variance (ANOVA) was used (Li et al., 2020). Prior to performing ANOVA, we strictly verified the two key assumptions of normality and homogeneity of variances for all datasets. Normality of the data distribution was tested using the Shapiro–Wilk test, and homogeneity of variances was assessed via the Levene’s test. For datasets that satisfied both assumptions (*P*> 0.05), one-way ANOVA was performed followed by Tukey’s honestly significant difference (HSD) test for post-hoc multiple comparisons. For datasets that violated the assumptions, the non-parametric Kruskal–Wallis H test was used instead, with the Dunn’s test for post-hoc analysis. In addition, redundancy analysis was performed using the RDA function in the vegan package of R software to analyze the explanatory power of environmental factors (physical and chemical parameters) on microbial community variation. Correlations between microbial genera, volatile compounds, and physicochemical parameters were analyzed using Spearman correlation coefficients. To avoid false positive results caused by multiple comparisons, the raw *P*-values were adjusted using the false discovery rate (FDR) method (Verhoeven et al., 2005). The correlation results were visualized using the corrplot and pheatmap packages in R software, with statistically significant correlations defined as adjusted *P*< 0.05.

## 3 Results

### 3.1 Physical properties and chemical compositions of sorghum

Table 2 provides a summary of the physical characteristics of the three sorghum varieties utilized in this research. A high breakdown level indicates that starch granules are more readily utilized by microorganisms after collapse (Wang, 2021). The disintegration values of starch in *Hongyingzi* sorghum (972.40 cP) and *Hei’e* sorghum (774.20 cP) were significantly higher than that of *Shandong* sorghum (213.00 cP) (*P*< 0.05). The setback viscosity value reflects the degree of aging of the starch during the cooling process. A higher setback viscosity level indicates a greater increase in viscosity of starch during aging, which is not conducive to microbial utilization (Wang, 2021). Additionally, significant differences in regain values were observed, with *Hongyingzi* sorghum (647.80 cP) and *Hei’e* sorghum (752.20 cP) showing lower regain values compared to that of *Shandong* sorghum (1414.60 cP) (*P*< 0.05).

**Table 2:** The gelatinization characteristics of sorghum.

Name	*Hongyingzi* sorghum		*Hei’e* sorghum	*Shandong* sorghum
Peak viscosity	2059.60 ± 6.80 ^b^	2104.20 ± 15.01 ^a^		1190.00 ± 11.05 ^c^
Hot-paste viscosity	1087.20 ± 9.42 ^b^	1330.00 ± 13.82 ^a^		977.00 ± 13.23 ^c^
Break down	972.40 ± 8.38 ^a^	774.20 ± 4.15 ^b^		213.00 ± 3.00 ^c^
Final viscosity	1735.00 ± 11.83 ^c^	2082.20 ± 26.30 ^a^		2391.60 ± 20.79 ^b^
Setback viscosity	647.80 ± 14.46 ^c^	752.20 ± 20.47 ^b^		1414.60 ± 17.36 ^a^
Peak time	4.60 ± 0.05 ^c^	5.48 ± 0.03 ^a^		5.03 ± 0.04 ^b^
Pasting temperature	76.69 ± 0.39 ^b^	75.23 ± 0.10 ^c^		82.40 ± 0.39 ^a^

Values represent means ± SD (n = 3). a ∼ c indicates the significance of the difference between different groups for the same indicator (*P*< 0.05).

The total starch content of the three sorghums varieties was approximately 70% and did not differ significantly (Figure S1E). *Shandong* sorghum exhibited the highest amylose percentage at 32.36%, which was significantly greater than those of the other two varieties (*P*<0.05) (Figure S1F). There were no significant differences in amylopectin content between *Hongyingzi* sorghum (77.83%) and *Hei’e* sorghum (74.23%), while both were significantly higher than that of *Shandong* sorghum (67.64%). Tannin contents of *Hongyingzi* sorghum and *Hei’e* sorghum were not significantly different (1.19 to 1.21 g/100 g) (*P*>0.05), which were significantly higher than that of *Shandong* sorghum (0.05 g/100 g) (*P*<0.05) (Figure S1D).

### 3.2 Physicochemical properties of fermented grains

**Fig. 1:**
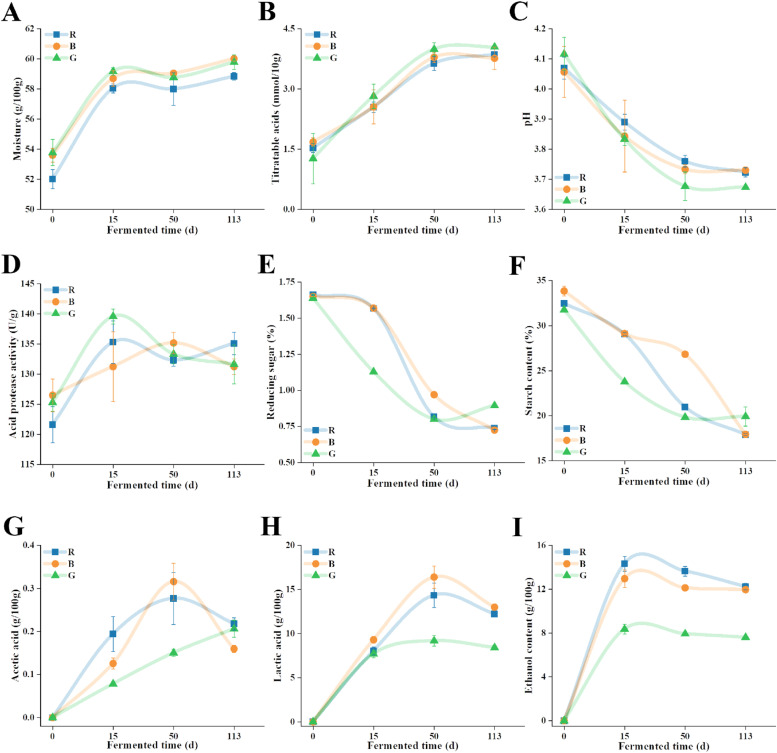
The evolution of physicochemical properties of fermented grains during the fermentation. (A) moisture. (B) titratable acid. (C) pH. (D) acid protease activity. (E) reducing sugar. (F) starch content. (G) acetic acid. (H) lactic acid. (I) ethanol content. R denotes the fermented group with *Hongyingzi* sorghum; B denotes the fermented group with *Hei’e* sorghum; G denotes the fermented group with *Shandong* sorghum.

The variation of physicochemical factors in three groups of fermented grains was investigated, and the results are shown in Fig. 1. Overall, the moisture of the fermented grains increased and then remained high (above 58 g/100 g) as the fermentation progressed to its final stage (Fig. 1A). The moisture affects the gelatinization process of sorghum starch and limit the water activity required for microbial growth, thus adversely affecting the quality of *Baijiu* (Zeng et al., 2021).

The metabolic activity of microbial communities during fermentation can be effectively monitored through key biochemical parameters, including pH levels, titratable acidity, and organic acid concentrations. As fermentation proceeded, titratable acidity index exhibited a progressive elevation pattern. The acidity of the fermented grains fluctuated between 1.27 and 4.05 mmol/10 g (Fig. 1B). Lactic acid, the key organic acid in fermentation, along with acetic acid, reached peak levels on the 50th day of fermentation in all three sorghum groups (Fig. 1H & G), indicating maximal microbial activity at this stage.

As the fermentation progressed, the levels of reducing sugar (Fig. 1E) and starch (Fig. 1F) significantly decreased in fermented grains. By the end of the fermentation, the reducing sugar in *Hongyingzi* fermented grains (Fig. 1E) had decreased to a low level (0.82 g/100 g) and stabilized, while the starch content in *Hei’e* fermented grains dropped sharply (Fig. 1F). In contrast, *Shandong* fermented grains showed less starch consumption (Fig. 1F). This might be attributed to the fact that the pH of the fermented grain at this stage was significantly lower than that of the other two sorghum varieties. The low pH inhibited the activity of enzymes such as amylase, leading to a substantial reduction in the rate of starch degradation. Meanwhile, the fermented grain of *Shandong* sorghum group contained a high abundance of *Candida* at the late stage of fermentation (Fig. 3B). Previous research has shown that the growth and metabolic characteristics of *Candida* can promote an increase in starch content through inhibiting starch degradation and contributing starch-like substances (Zeng et al., 2024). Additionally, the ethanol content increased significantly as fermentation progressed, rising rapidly by 8.5% (w/w) within the first 15 days of fermentation (Fig. 1I).

### 3.3 Dynamic distribution of volatile compounds during fermentation of sorghum

**Fig. 2:**
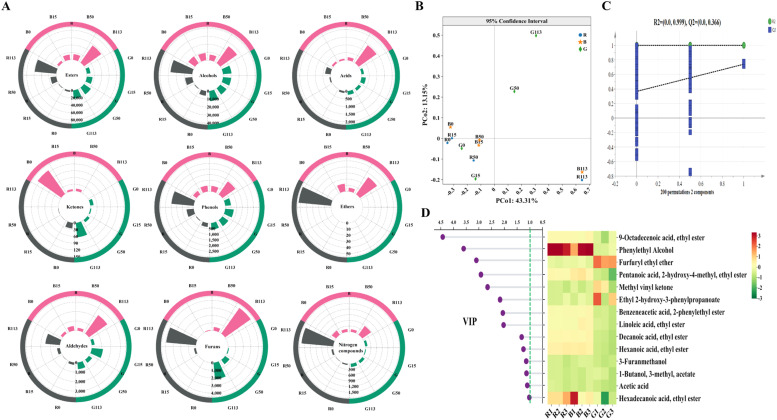
Analysis of volatiles. (A) Circle plot of changes in the flavor compounds during the fermentation. (B) Principal Coordinate Analysis (PCoA) between volatile compounds (X variables) and fermented grain samples (Y variables). (C) Analysis of indicator volatile compounds based on the OPLS-DA model. (D) Compounds with VIP > 1 (screened via the OPLS-DA model). R denotes the fermented group with *Hongyingzi* sorghum; B denotes the fermented group with *Hei’e* sorghum; G denotes the fermented group with *Shandong* sorghum.

A total of 194 volatile compounds were identified across the three groups of fermented grains, including 84 esters, 18 alcohols, 12 phenols, 16 aldehydes and ketones, 17 acids, 3 ethers, 8 furans, 25 nitrogen compounds and 11 other compounds (Fig. 2A). Overall, alcohols and esters were the dominant flavor substances in the middle and late stages of fermentation. The levels of alcohols and esters accumulated in *Hongyingzi* and *Hei’e* fermented grains were comparable, while both being significantly higher than those in *Shandong* fermented grains (Fig. 2A). In addition, there were significant differences in the flavor profiles among the three groups of fermented grains during the middle and late stages of fermentation (Fig. 2B), likely due to the varying rates of microbial metabolic activity in each group.

To further investigate the marker metabolites influencing volatile profiles during fermentation, we constructed a supervised PLS-DA model. The model’s parameters, specifically R^2^ =0.999 and Q^2^ = 0.36 were employed to assess its stability and predictive capabilities (Fig. 2C). As shown in Fig. 2D, hexadecenoic acid ethyl ester, phenylethyl alcohol, linoleic acid ethyl ester, decanoic acid ethyl ester, hexanoic acid ethyl ester, benzeneacetic acid 2-phenylethyl ester were identified as the volatile metabolites that produced significant differences in the fermentation of the three sorghum varieties. Hexadecenoic acid ethyl ester reduces the ethanol sharpness and contributing to the mellow taste (Bian et al., 2025). It also serves as a precursor to γ-nonalactone, which balances roasted notes in the “fried sesame” aroma. Phenylethyl alcohol imparts rose-like floral notes. It could react with acetic acid to form phenethyl acetate, amplifying the prominent roasted aroma (Xu et al., 2023). A total of 197 compounds were identified in the SFB samples in full scan mode. The composition of volatiles in SFB samples fermented from three sorghum varieties was different (Figure S2). They were subjected to Random Forest Analysis to screen the three SFB samples for characteristic flavors (Mean Decrease Accuracy > 2.5), with the largest difference in volatile content between *Hongyingzi* sorghum and *Shandong* sorghum (Table S1). A variety-specific shift in these differential volatiles was observed during fermentation. For example, 9-octadecenoic acid ethyl ester accumulated steadily in *Hei’e* and *Shandong* sorghum fermented grains during the initial 15 days but declined progressively in *Hongyingzi* sorghum fermentation. These findings suggest that the development and accumulation of flavor profiles in fermented grains are potentially related to the variety of the raw material used.

### 3.4 Microbial diversity and community succession during fermentation

**Fig. 3:**
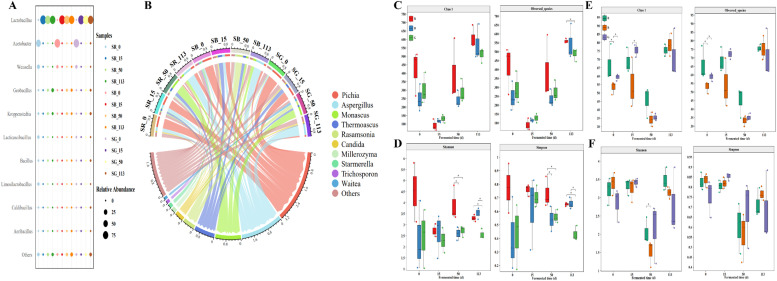
Succession of the bacterial/fungal community during the fermentation of three groups fermented grain. (A) The circos landscape shows the relative abundance of bacterial genus level amongst the different fermented grains during the fermentation. (B) The circos landscape shows dynamics in the fungal community at the genus level with a relative abundance of >1%. (C) Box plots of the changes in bacterial richness (Chao1 index and Obversed_species) during the fermentation. (D) Box plots of the changes in bacterial diversity (Shannon index and Simpson index) during the fermentation. (E) Box plots of the changes in fungal richness (Chao1 index and Obversed_species) during the fermentation. (F) Box plots of the changes in fungal diversity (Shannon index and Simpson index) during the fermentation. R denotes the fermented group with *Hongyingzi* sorghum; B denotes the fermented group with *Hei’e* sorghum; G denotes the fermented group with *Shandong* sorghum. * denotes *P*< 0.05.

The bacterial richness and diversity index during the fermentation are illustrated in Fig. 3C & D, respectively. Compared to *Hei’e* and *Shandong* fermented sorghums, *Hongyingzi* fermented sorghum showed higher bacterial community diversity and richness throughout the fermentation period. The Kruskal-Wallis test and Duncan’s test showed that at the end of fermentation, *Hongyingzi* fermented grain showed similar bacterial community diversity and richness to *Hei’e* fermented grain, with both differing significantly from *Shandong* fermented grain. Changes in microbial communities β-diversity at the end of fermentation in three varieties of sorghum also indicated the similarity of microbial structure in *Hongyingzi* and *Hei’e* fermented grains (Figure S3).

At the beginning of fermentation, *Acetobacter* and *Weissella* were the dominant bacterial genus in all three groups of fermented grains, with an average relative abundance of >50%. However, from the 15th day of fermentation until the end, *Lactobacillus* maintained the absolute dominance.

Quantitative assessments of fungal community complexity throughout the fermentation process are illustrated in Fig. 3E & F, presenting both species richness and diversity indices. After fermentation completion, there were no significant differences in the overall diversity and richness of fungal communities among the three groups of fermented grains. The richness of fungal communities in all three groups reached its lowest level by the 15th day of fermentation and peaked on the 113th day. All fungal ASVs were annotated at the genus levels (Fig. 3B) to investigate the community succession during fermentation. At the beginning of the fermentation, the dominant fungi in *Hongyingzi* sorghum were *Pichia* and *Aspergillus*, whereas the dominant fungus in other varieties of sorghum was only *Pichia*. During the early fermentation stage, *Aspergillus* exhibit a rapid growth rate, enabling them to rapidly utilize nutrients from raw materials for massive proliferation, occupy dominant ecological niches in fermented grain, and inhibit the growth of spoilage bacteria (e.g., *Escherichia coli*). In addition, organic acids such as acetic acid secreted by *Aspergillus* can reduce the pH of fermented grain, thereby suppressing the growth of acid-intolerant miscellaneous bacteria. Furthermore, bioactive metabolites (e.g., B-group vitamins) synthesized by *Aspergillus* can promote the growth and reproduction of yeasts, which is conducive to ethanol production (Liu et al., 2025). By the 15th day of fermentation, significant differences in fungal species were observed among the three groups of fermented grains. *Pichia* remained the dominant fungus in all three fermented grains, alongside an increasing abundance of *Thermoascus*, which also became dominant in the fermented grains of *Hei’e* and *Shandong* groups. By the end of the fermentation, *Aspergillus* reemerged as the dominant fungus in all three groups.

### 3.5 Interactions of microbial communities during fermentation

**Fig. 4:**
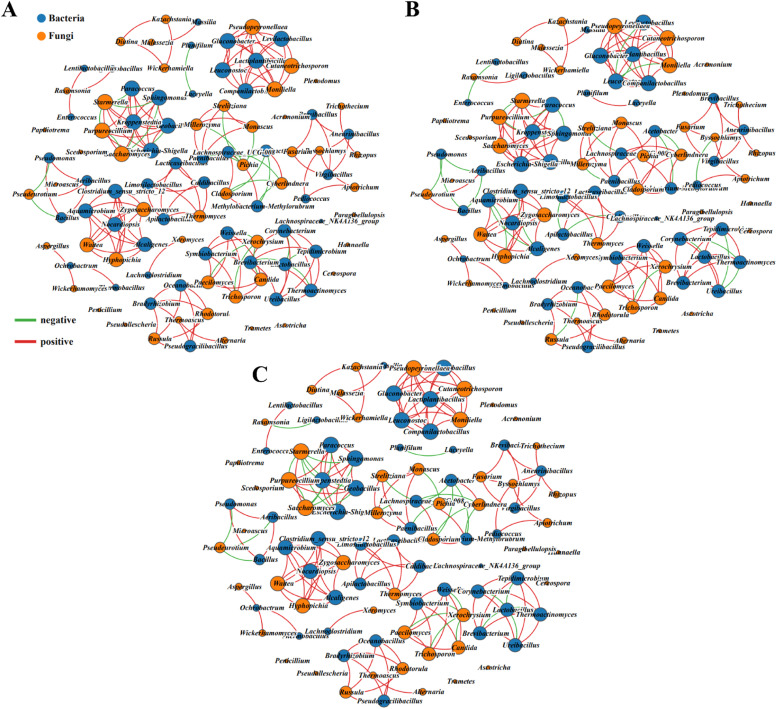
Bacterial-fungal interactions during fermentation of three fermented grains. (A) *Hongyingzi* sorghum. (B) *Hei’e* sorghum. (C) *Shandong* sorghum.

The interactions between bacteria and fungi during the fermentation of fermented grains were further analyzed by constructing cross-domain networks (Tan et al., 2021). As shown in Fig. 4, there were 174, 220 and 249 pairs of bacterial-fungal connections in *Hongyingzi, Hei’e* and *Shandong* sorghum, respectively. Although *Shandong* group possessed a larger bacterial-fungal interactions network, there were 50 negative bacterial-fungal correlations, which accounting for >20% of the total cross-domain connections. Nevertheless, negative bacterial-fungal correlations in *Hongyingzi* and *Hei’e* fermented grains accounted only for 16% and 16.82%, respectively. This indicated that *Shandong* fermented grain had more antagonistic or competitive relationships between bacterial and fungal species during the fermentation process (Kang et al., 2022a), and might have a negative effect on the fermentation. However, the bacteria-fungi relationship in groups of *Hongyingzi* and *Hei’e* fermented grains were more similar and exhibited more cooperative and symbiotic relationships.

### 3.6 Physicochemical drivers of microbial community structure

To identify the key determinants governing microbial community dynamics, a Mantel test was employed to assess the potential associations between bacterial and fungal population structures, multiple physicochemical parameters, and volatile organic compound profiles (Figure S3). The bacterial community showed highly significant and strong correlations with lactic acid and ethanol content. In addition, it also had strong positive correlations with acidity, pH, reducing sugar, starch, and moisture. Fungal communities showed strong correlations with all environmental factors except moisture and acid protease activity. In contrast, the volatile metabolite profile was significantly correlated with reducing sugar and starch content. In addition, the bacterial diversity and structure, positively and significantly affected by lactic acid and ethanol content, play a stronger and positive role in shaping volatile metabolites.

On the other hand, it revealed that the bacterial community was significantly affected by a greater number of physicochemical factors compared to the fungal community. For example, pH exerted a more pronounced impact on bacterial communities, whereas its effect on fungal communities was relatively minor. Bacterial cell membranes were directly exposed to the external environment and lack the chitinous protective layer unique to fungal cell walls, rendering them more sensitive to pH fluctuations (Cheng et al., 2024).During the fermentation of the fermented grain, when the pH dropped sharply from 4.0 to 3.7, *Lactobacillus* rapidly became the dominant bacterial genus due to its acid-producing and acid-tolerant properties, directly triggering a drastic succession of the bacterial community structure, where *Lactobacillus* replaced *Acetobacter* that dominated the initial fermentation stage. In addition, regarding moisture content, a significant correlation was observed between bacterial communities and moisture content, while no such correlation exists for fungal communities. During the fermentation of the fermented grain, with the moisture content ranging from 52% to 60%, water filled the pores of the fermented grain, resulting in poor oxygen permeability. This condition inhibited the growth of certain aerobic bacteria such as *Bacillus*. In contrast, the mycelial structure of fungi conferred stronger water absorption and retention capabilities. Previous research (Park et al., 2025) had reported that the mycelia of *Aspergillus* can still grow slowly under low water activity conditions (aw = 0.8), which was far superior to the tolerance of bacteria. It suggests that bacteria exhibit higher sensitivity to variations in the fermentation environment and the differences in microbial communities across different groups of fermented grains may primarily be driven by bacterial genera.

**Fig. 5:**
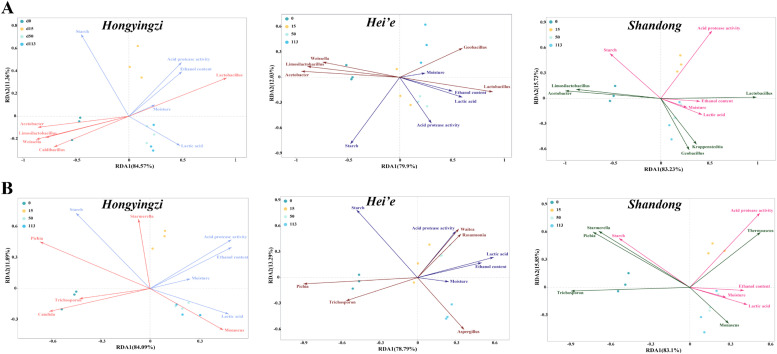
Redundancy analysis (RDA) for the dominant genera and fermentation parameters. (A) Dominant bacterial genera. (B) Dominant fungal genera.

To further identify factors driving changes in the dominant flora, we performed the redundancy analysis. The results showed that the correlation between microbial communities and physicochemical factors varied depending on the sorghum varieties. In fact, for the bacterial community, *Weissella* was associated with physicochemical factors such as starch, moisture, and lactic acid in both *Hongyingzi* and *Hei’e* fermented grains, but not in *Shandong* group (Fig. 5A). For the fungal community, ethanol content positively correlated with *Starmerella* in *Hongyingzi* fermented grains, but did not correlate with *Starmerella* in *Hei’e* fermented grains and negatively correlated with *Starmerella* in *Shandong* fermented grains (Fig. 5B).

### 3.7 Effect of abiotic factors on the flavor profiles during fermentation

**Fig. 6:**
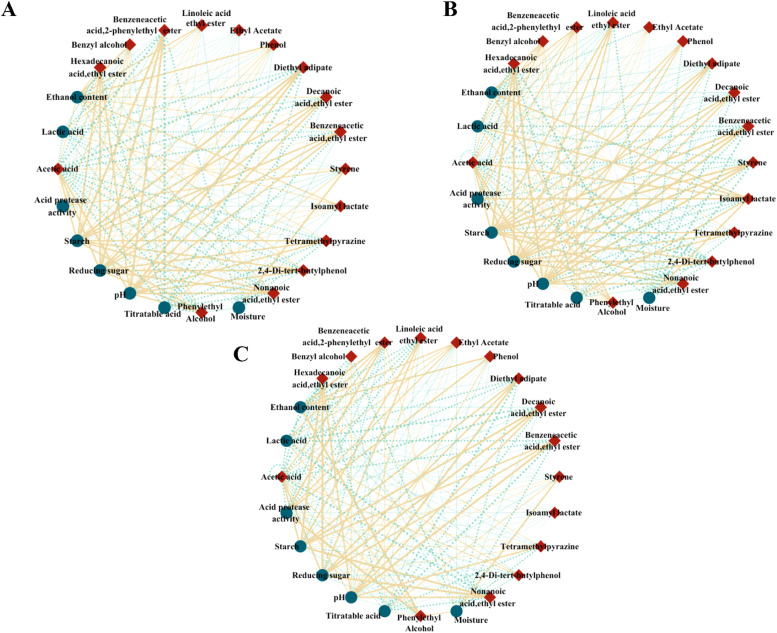
A correlation network between abiotic factors and flavor compounds during the fermentation of fermented grain. The blue circles represent abiotic factors; the red diamonds represent flavor compounds. The blue dotted line shows a positive correlation, and the solid yellow line shows a negative correlation. (A) *Hongyingzi* sorghum. (B) *Hei’e* sorghum. (C) *Shandong* sorghum.

To evaluate the influence of non-living environmental variables on the production of essential aromatic substances, a Spearman’s rank correlation coefficient was generated. As shown in Fig. 6, styrene exhibited a positive correlation with reducing sugar (r = 0.4, *P*= 0.6) and starch content (r = 0.4, *P*= 0.6) in *Hongyingzi* fermented grains. In contrast, it exhibited significant negative correlations with reducing sugar content (r = −0.6, *P*= 0.03) and starch content (r = −0.4, *P*= 0.07) in the fermented grains of *Hei’e*. The fermented grain of *Shandong* displayed a similar correlation pattern to those of *Hei’e*, with negative correlations observed between styrene and reducing sugar content (r = −0.6, *P*= 0.2) as well as starch content (r = −0.5, *P*= 0.02). Additionally, benzyl alcohol was positively correlated with alcohol content in the fermented grain of *Shandong* (r = 0.6, *P*= 0.64), whereas negative correlations were found in the fermented grains of *Hongyingzi* (r = −0.4, *P*= 0.6) and *Hei’e* (r = −0.6, *P*= 0.6). These results indicate that the correlation between the physicochemical factors and volatile compounds in fermented grains varied with sorghum varieties.

### 3.8 Correlation analysis between microbial community and flavor compounds

**Fig. 7:**
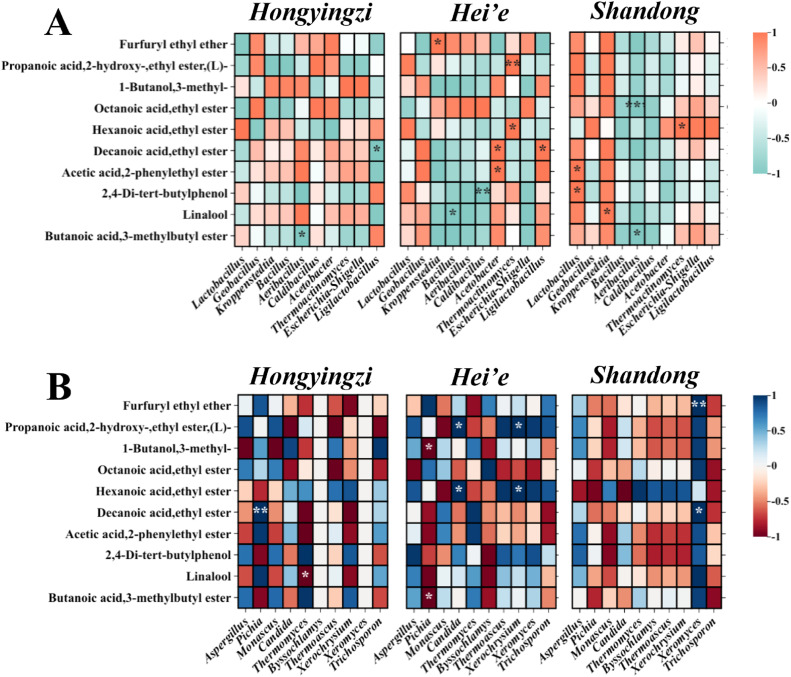
Correlations between dominant microbiota genera and flavor compounds at the end of fermentation. (A) Bacterial genera. (B) Fungal genera.

The Pearson correlation coefficients was conducted to illustrate the relationship between the dominant microbial communities and flavor compounds in SFB (shown in Fig. 7A & B). A total of seven bacterial and five fungal genera had significant effects on flavor compounds (r > 0.7 and *P*< 0.05).

The correlation results showed that bacterial genera *Thermoactinomyces, Kroppenstedtia, Acetobacter* and *Lactobacillus* had a significantly positive correlation with furfuryl ethyl ether, propanoic acid, 2‑hydroxy-, ethyl ester, linalool, hexanoic acid, ethyl ester and decanoic acid, ethyl ester (r>0.9 and *P*<0.05), whereas *Aeribacillus, Ligilactobacillus, Bacillus* and *Caldibacillus* were negatively corelated with butanoic acid, 3-methylbutyl ester, decanoic acid, ethyl ester, linalool, 2,4-Di‑tert-butylphenol and octanoic acid, ethyl ester (r<−0.9 and *P*<0.05). For fungus, *Xeromyces, Pichia, Candida* and *Xerochrysium* showed a positive correlation (r>0.9 and *P*<0.05) with decanoic acid, ethyl ester, furfuryl ethyl ether and hexanoic acid, ethyl ester, whereas *Pichia* and *Thermomyces* showed a negative correlation with linalool, butanoic acid, 3-methylbutyl ester and 1-butanol,3-methyl (r<−0.9 and *P*<0.05).

Changes in fungal communities in *Shandong* sorghum fermentation had the least effect on volatile expression, and the microbial communities in *Hei’e* sorghum fermentation had the greatest effect on volatile expression. In addition, the correlations between microbial communities and volatiles in fermented grains differed across varieties of sorghum. For example, linalool, which constitutes the main flavor of SFB, showed a positive correlation with *Bacillus* in *Hongyingzi* sorghum fermentation while a negative correlation in *Hei’e* sorghum and *Shandong* sorghum fermentations. Decanoic acid, ethyl ester and *Pichia* were significantly positively correlated in *Hongyingzi* sorghum fermentation while were negatively correlated in *Hei’e* sorghum and *Shandong* sorghum fermentation.

## 4 Discussion

In the traditional fermentation process of *Baijiu*, raw materials play crucial roles, which not only influencing the structure of the core microbial community, but also determining the production of volatile compounds. However, there is a lack of insight into how raw materials specifically affect the microbial composition and their functions during SFB fermentation. Therefore, this study was devoted to investigating the microbial community composition, diversity changes, and microbial succession rules of different varieties of sorghum during SFB fermentation.

### 4.1 Physical and chemical factors of sorghum affect microbial communities

We examined the physicochemical composition of the sorghum and the microbial community during the fermentation of the fermented grains, respectively. Various physicochemical factors, such as starch content, reducing sugars, and acidity, can influence the assembly of microbial communities during fermentation. We extended these findings to show that the main physicochemical factors affecting the construction of the microbiome can vary depending on the sorghum varieties (Fig. 5). Different sorghum varieties contain varying levels of starches, proteins, and polyphenols. These compounds directly influence the fermentation process. They act as substrates for microbial growth. Their availability can affect the types of microorganisms that dominate the fermentation. Higher starch content supports the growth of saccharifying and alcohol-producing microbes. In contrast, variations in proteins and polyphenols influence the development of flavor compounds. In this study, the amylopectin content of *Hongyingzi* sorghum was significantly higher than that of *Hei’e* sorghum and *Shandong* sorghum (*P*< 0.05) (Figure S1F). Amylopectin, which has more short-chain branches, is easily decomposed into glucose by amylase; thus, the sorghum with elevated amylopectin levels could supply a greater quantity of reducing sugar at the beginning of fermentation, thereby supporting enhanced microbial growth and metabolic activity. This biochemical mechanism likely accounted for the observed higher reducing sugar content in *Hongyingzi* sorghum compared to other sorghum varieties in the early stages of fermentation. (Fig. 1E). In addition, total starch content, had no significant influence on fungal assembly in *Hei’e* sorghum fermentation, but it did affect the fungal communities in the other fermented sorghum varieties. The differences in the starch composition of sorghum varieties (Wang et al., 2021b), resulting in different effects on fungal genera growth. The specific amylose and amylopectin ratios, along with the structure and availability of starch, could influence how different fungal communities metabolize these carbohydrates, ultimately affecting their growth and succession during the fermentation process. Interestingly, *Aspergillus* is known for its ability to secrete enzymes such as amylase, protease, and glucoamylase, which contribute significantly to the growth of fermented microorganisms (Wang et al., 2023). *Shandong* sorghum exhibited lower protease activity and starch consumption after fermentation than the other two varieties of sorghum. This is consistent with the microbial community analysis, which revealed that *Shandong* sorghum had a lower abundance of *Aspergillus* after fermentation. This divergence underscores the critical interplay between the specific physicochemical properties (e.g., tannin levels, gelatinization enthalpy) of sorghum and the microbial community, which collectively shape metabolic efficiency and flavor compound synthesis.

### 4.2 Contributions of physicochemical factors to flavor

The significant differences in flavor profiles among the fermented grains highlight the crucial role of raw materials in determining the final taste of SFB. The consistent flavor profiles in *Hongyingzi* and *Hei’e* sorghums, compared to the distinct profile of *Shandong*, suggest that the nutrient compositions of the sorghums influence the metabolic pathways and microbial interactions responsible for flavor development. Tannins can directly influence the flavor profile of *Baijiu* by interacting with proteins and polysaccharides, contributing to bitterness and astringency. While *Baijiu* is distilled, certain tannin derivatives or their influence on microbial metabolites might still affect the final product’s sensory characteristics. Higher tannin content in *Hongyingzi* and *Hei’e* sorghums, could enhance the astringency of the fermented grains, which may result in a sharper and more robust flavor profile. At the same time, tannin can undergo decomposition by certain bacteria (Liu et al., 2006), resulting in the production of odorous phenols such as 4-ethyl-2-methoxyphenol, which contribute to distinct smoky, woody, and clove-like spicy flavors to *Baijiu* and this compound is a key contributor to the phenolic and medicinal nuances in SFB (Liu et al., 2006; Xu et al., 2018). As shown in Figure S1F, tannin content was significantly higher in *Hongyingzi* and *Hei’e* sorghum compared to *Shandong* sorghum. A parallel trend was observed for 4-ethyl-2-methoxyphenol (4-ethylguaiacol), with its content also being elevated in *Hongyingzi* and *Hei’e* fermented grains. Decanoic acid ethyl ester is one of the key flavor esters of SFB, and it contributes significantly to the creamy aroma of SFB (Zheng et al., 2016). Decanoic acid ethyl ester can also synergize with 4-ethylguaiacol and other substances to form a “sesame flavor” characteristic. As shown in Fig. 7, there is a negative correlation between decanoic acid, ethyl ester and the abundance of *Ligilactobacillus*. The lower abundance of *Ligilactobacillus* in *Hongyingzi* sorghum is consistent with its high content of decanoic acid ethyl ester.

### 4.3 Different sorghum varieties lead to microbial differences in SFB fermentation

Within the confined environment of the fermentation pit, varying selective pressures, such as temperature, pH, oxygen availability, and substrate composition, likely account for the distinct microbial succession patterns observed in the fermented grains and their subsequent influence on volatile compound production. Interestingly, apart from *Lactobacillus*, the dominant microbes in fermented grains varied between studies. A previous study identified *Lactobacillus, Weissella, Acetobacter, Pediococcus, Pichia*, and *Candida* are dominant microbial communities (Ji et al., 2023). However, *Pediococcus* was not the dominant flora in our study. The observed difference in microbial composition and succession patterns could be attributed to variations in microbial niches shaped by differences in raw materials. Significant differences in tannin acid content were identified in the three varieties of sorghum. Tannin has a regulatory effect on the growth of ester-producing microbial flora (Xu et al., 2018), as evidenced by differences in microbial interactions during the fermentation. Higher tannin content, such as in *Hongyingzi* and *Hei’e* sorghum, may inhibit certain microbial species, particularly bacteria, while selectively promoting tannin-tolerant microorganisms. This selective pressure can alter the microbial composition and interactions, leading to different fermentation dynamics. This could result in variations in the production of key flavor compounds, such as esters and alcohols, due to the influence on metabolic pathways. Consequently, sorghum varieties exerted an indirect influence on the composition and dynamics of the microbial community by regulating the physicochemical properties of the fermented grains.

Fermented foods typically originated from raw materials that contain local microbial communities. In the production of solid-state fermented *Baijiu*, after steaming, three varieties of sorghum naturally accumulate a certain number of microorganisms during the initial stage of stack fermentation. Although the dominant species of the initial microbial communities of the three varieties of the fermented grains were the same, the proportion of dominant species varied. Previous study showed that different fermenting species exhibit varying dynamic characteristics during fermentation (Bittleston et al., 2020), manifested by differences in microbial succession rates in SFB production. And the abundance of species in the complex community directly determines their functions (Rivett and Bell, 2018). The observed differences suggest that different sorghum varieties may influence the metabolite profiles of the fermentation system by selectively shaping the structure of specific microbial communities, which in turn affects the flavor of the fermentation system. Thermophilic bacteria (e.g., *Thermoactinomyces, Caldibacillus*) drive starch degradation and Maillard reactions at high temperatures, while acid-producing *Lactobacillus* and *Bacillus* modulate the balance of organic acids and aromatic esters. Fungal genera like *Xeromyces* and *Thermomyces* further enhance flavor complexity through lignocellulose breakdown and phenolic metabolism, illustrating the functional synergy of microbial consortia in shaping SFB’s sensory profile. These findings establish a framework for tailoring microbial consortia in SFB fermentation through strategic selection of raw materials-particularly sorghum cultivars with defined tannin and starch profiles-to preferentially enrich functional guilds like *Lactobacillus* and *Acetobacter*, thereby steering metabolic outputs toward desirable flavor consistency and product standardization.

## 5 Conclusion

In this study, we investigated the alcoholic fermentation of three varieties of sorghum and revealed how the microbial communities changed during fermentation. Our results suggest that the formation of flavor compounds in SFB involves intricate biochemical pathways that extend beyond microbial interactions alone. Key microbial genera, including bacteria (*Thermoactinomyces, Lactobacillus, Kroppenstedtia, Aerbacillus, Acetobacter*) and fungi (*Xeromyces, Pichia, Candida, Xerochrysium*), are critical contributions to the synthesis of flavor compounds in SFB. Both *Hongyingzi* and *Hei’e* sorghums exhibited similar flavor profiles by the end of fermentation, showing a significant contrast with *Shandong* sorghum, which may be attributed to the physicochemical properties of sorghum. The differences in physicochemical composition affect the assembly of microbiota, and that interactions between bacteria and fungi during fermentation affect microbial succession rates, ultimately shaping the flavor profile of the fermented grains. In summary, this research offers fundamental insights into the influence of sorghum variety on the fermentation dynamics of SFB and the subsequent development of flavor profiles. It emphasizes the critical role of raw materials in shaping microbial communities and flavor profiles.

## Funding statement

This work was supported by10.13039/100012542Sichuan Science and Technology Program (Grant No 2025ZNSFSC0228), the 10.13039/501100001809National Natural Science Foundation of China (Grant No 32272275), and the 10.13039/501100015714Science and Technology Talents and Platforms Program of Yunnan Province (Grant No 202305AF150210).

## Data availability statement

The data presented in the study are deposited in the NCBI Sequence Read Archive (SRA) repository, accession number (PRJNA1168063).

## CRediT authorship contribution statement

**Meina Jiang:** Data curation, Formal analysis, Methodology, Validation, Visualization, Writing – original draft, Writing – review & editing. **Jiaxin Tang:** Data curation, Formal analysis, Writing – review & editing. **Angui Lai:** Conceptualization, Writing – review & editing. **Wei Sun:** Conceptualization, Writing – review & editing. **Xue Liu:** Data curation, Formal analysis, Investigation. **Zhichao Shang:** Data curation, Formal analysis, Investigation. **Zuzhong Zheng:** Formal analysis, Funding acquisition. **Xiaoxue Chen:** Writing – review & editing. **Yansong Xue:** Conceptualization, Funding acquisition, Supervision, Project administration, Writing – review & editing. **Bei-Zhong Han:** Conceptualization, Funding acquisition, Writing – review & editing.

## Declaration of competing interest

The authors declare that they have no known competing financial interests or personal relationships that could have appeared to influence the work reported in this paper.

## Appendix Supplementary materials

Image, application 1

Image, application 2

Image, application 3
