# Supplementary material for: Multi-omics analysis-based insights into the microbial community composition and flavor development potentiality of different varieties of sorghum (Sorghum bicolor L. Moench) fermented into Sesame flavor Baijiu
Source: Curr Res Microb Sci. 2026 May 15;10:100606. doi: 10.1016/j.crmicr.2026.100606 (PMC13213687; doi:10.1016/j.crmicr.2026.100606)
Supplement: Supplementary file 1 [file mmc1.pdf]

Authorship change request

Important information. Please read before completing this form.
This form is to request any change in authorship (additions, removals, or reordering) after the submission of a manuscript, including changes in corresponding authors, if any. This form should not be used for changes requested *after* publication or for name changes or corrections.

Prior to completing this form, all authors should carefully review the 'Duties of Authors' section of the Elsevier publishing ethics policy, and in particular, the sections on:

- Authorship of the paper
- The use of generative AI and AI-assisted technologies in scientific writing and in figures, images and artwork

Please also carefully review the journal's guide for authors (this might also be referred to as 'instructions for authors') as some journals may have additional authorship criteria (e.g., the ICMJE guidelines for authorship).

The publisher and editor cannot investigate or mediate any authorship disputes. If you are unable to obtain agreement from all authors, including those you intend to remove, we recommend seeking guidance from your institution. We will not consider your change request and will not proceed with the publication of your manuscript until all outstanding authorship disputes are resolved.

If your manuscript is still under consideration, this completed form should be submitted in Editorial Manager for consideration as part of your revision submission (use the 'cover letter' file type).

If your manuscript has already been accepted and is in the proofing or production stages, please return this completed form to the Journal Manager.

If the final version of your manuscript has already been published, a corrigendum will be required. Please see the Article Correction, Retraction and Removal Policy.

Section 1. Submission information

To be completed by the corresponding author.

**Submission information**	
**Journal title**	Current Research in Microbial Sciences
**Manuscript number and/or article number**	CRMICR-D-25-00478
**Manuscript title**	Multi-omics analysis-based insights into the microbial community composition and flavor development p

Change(s) requested (indicate as appropriate)

☑ Add new author(s)	☐ Remove author(s)
☐ Change the corresponding author	☑ Change the order of authors

Section 2. Author(s) added or removed

Complete one table for each author to be added or removed. Please include as much detail as possible in the "Reason for change" section so that we can evaluate if the change is necessary. At a minimum, this should include an explanation for why the change is being requested and why the author was/was not included in the original author list.

If the form is not provided, incomplete, or the reasons provided are insufficiently detailed or do not address the points above, your request will be denied and your submission may be rejected.

Unless instructed by the editor, further changes to the author list without an approved authorship change request will result in the rejection of your submission, or retraction, if the article has already been published.

2.1 Author information

**Given/first name(s)**	Zhichao
**Family/last name**	Shang
**Email address**	2823124158@qq.com
**Institution**	Shandong Jing Co., Ltd.
**Change(s) requested** (indicate as appropriate)	☐ Add new author ☐ Remove author ☐ Make the corresponding author

Individual contributions per CRediT Contributor Roles Taxonomy (required for author additions only)

☐ Conceptualization	☑ Data curation	☑ Formal analysis
☐ Funding acquisition	☑ Investigation	☐ Methodology
☐ Project administration	☐ Resources	☐ Software
☐ Supervision	☐ Validation	☐ Visualization
☐ Writing – original draft	☐ Writing – review & editing	

Reason for the change

We sincerely apologize for the oversight of omitting Zhichao Shang from the author list in the initial submission of our manuscript.
We hereby formally requests to add Zhichao Shang (Affiliation: Shandong Jingzhi Co., Ltd., Email: 2823124158@qq.com) as a co-author of this study. The newly added author made substantial contributions to the work, specifically undertaking the following key tasks corresponding to the CRediT author contribution taxonomy:
Data curation: Responsible for standardizing the raw data of volatile compound detection;
Formal analysis: Performed ANOVA with Turkey HSD test statistical analysis;
Investigation: Participated in the on-site fermentation experiments of different sorghum varieties and sample collection in the industrial workshop.
All existing authors have reviewed and unanimously approved the addition of Zhichao Shang to the author list. The newly added author has also confirmed their consent to be listed as a co-author and approved the revised content of the manuscript.

2.2 Author information

**Given/first name(s)**	
**Family/last name**	
**Email address**	
**Institution**	
**Change(s) requested** (indicate as appropriate)	☐ Add new author ☐ Remove author ☐ Make the corresponding author

Individual contributions per CRediT Contributor Roles Taxonomy (required for author additions only)

☐ Conceptualization	☐ Data curation	☐ Formal analysis
☐ Funding acquisition	☐ Investigation	☐ Methodology
☐ Project administration	☐ Resources	☐ Software
☐ Supervision	☐ Validation	☐ Visualization
☐ Writing – original draft	☐ Writing – review & editing	

Reason for the change

2.3 Author information

**Given/first name(s)**	
**Family/last name**	
**Email address**	
**Institution**	

Change(s) requested (indicate as appropriate) ☐ Add new author ☐ Remove author ☐ Make the corresponding author

Individual contributions per CRediT Contributor Roles Taxonomy (required for author additions only)

☐ Conceptualization	☐ Data curation	☐ Formal analysis
☐ Funding acquisition	☐ Investigation	☐ Methodology
☐ Project administration	☐ Resources	☐ Software
☐ Supervision	☐ Validation	☐ Visualization
☐ Writing – original draft	☐ Writing – review & editing	

Reason for the change

2.4 Author information

**Given/first name(s)**	
**Family/last name**	
**Email address**	
**Institution**	
**Change(s) requested** (indicate as appropriate)	☐ Add new author ☐ Remove author ☐ Make the corresponding author

Individual contributions per CRediT Contributor Roles Taxonomy (required for author additions only)

☐ Conceptualization	☐ Data curation	☐ Formal analysis
☐ Funding acquisition	☐ Investigation	☐ Methodology
☐ Project administration	☐ Resources	☐ Software
☐ Supervision	☐ Validation	☐ Visualization
☐ Writing – original draft	☐ Writing – review & editing	

Reason for the change

2.5 Author information

**Given/first name(s)**	
**Family/last name**	
**Email address**	
**Institution**	

Change(s) requested (indicate as appropriate) ☐ Add new author ☐ Remove author ☐ Make the corresponding author

Individual contributions per CRediT Contributor Roles Taxonomy (required for author additions only)

☐ Conceptualization	☐ Data curation	☐ Formal analysis
☐ Funding acquisition	☐ Investigation	☐ Methodology
☐ Project administration	☐ Resources	☐ Software
☐ Supervision	☐ Validation	☐ Visualization
☐ Writing – original draft	☐ Writing – review & editing	

Reason for the change

*Add additional page(s) as needed for more requested changes.

Section 3. Author order and agreement

Provide the author list in the order that you would like it to be published.

The form must be signed individually by each author, including any added/removed authors. In cases of consortia group authorship, the corresponding author may sign on behalf of the group.

While handwritten signatures are acceptable, we highly encourage the use of electronic signature software (DocuSign, Adobe Sign, HelloSign, or similar) with valid e-signatures. These signatures should reflect your institutional information and email, as provided in the author list below. **Typed signatures or images of signatures will not be accepted**.

By signing this form all authors agree:

1) that they have read and acknowledge the publishing ethics policies linked in the "Important Information" section of this form;
2) agree to the addition and/or removal of the authors listed in section 2 and to the revised order of the author list in this section 3, and;
3) that all information provided accurately reflects the authorship of the article.

Agreement of removed author(s)

Full name	Email address	Signature	Date

*Add additional page(s) as needed.

Proposed author list

Order	Full name	Email address	Signature	Date
01	Meina Jiang	mn.jiang@cau.edu.cn	Meina Jiang China Agricultural University mn.jiang@cau.edu.cn	2025.12.11
02	Jiaxin Tang	tangjx815@163.com	Jiaxin Tang China Agricultural University tangjx815@163.com	2025.12.11
03	Angui Lai	13606366779@139.com	Angui Lai Shandong Jingzhi Co., Ltd. 13606366779@139.com	2025.12.11
04	Wei Sun	990039630@qq.com	Wei Sun Shandong Jingzhi Co., Ltd 990039630@qq.com	2025.12.11
05	Xue Liu	xuechao205@163.com	Xue Liu Shandong Jingzhi Co., Ltd. xuechao@163.com	2025.12.11
06	Zhichao Shang	2823124158@qq.com	Zhichao Shang Shandong Jingzhi Co., Ltd. 2823124158@qq.com	2025.12.11
07	Zuzhong Zheng	zuzongzheng@qq.com	Zuzhong Zheng Yunnan Chishuiyuan Liquor Industry Co., Ltd. zuzongzheng@qq.com	2025.12.11
08	Xiaoxue Chen	chen.xx@cau.edu.cn	Xiaoxue. Chen China Agricultural University chen.xx@cau.edu.cn	2025.12.11
09	Yansong Xue	yansong.xue@cau.edu.cn	Yansong Xue China Agricultural University yansong.xue@cau.edu.cn	2025.12.11
10	Bei-Zhong Han	bz.han@cau.edu.cn	Bei-Zhong Han China Agricultural University bz.han@cau.edu.cn	2025.12.11
11				
12				
13				
14				
15				
16				
17				
18				
19				
20				
21				
22				
23				
24				
25				

*Add additional page(s) as needed.
