# Supplementary material for: Multi-omics analysis-based insights into the microbial community composition and flavor development potentiality of different varieties of sorghum (Sorghum bicolor L. Moench) fermented into Sesame flavor Baijiu
Source: Curr Res Microb Sci. 2026 May 15;10:100606. doi: 10.1016/j.crmicr.2026.100606 (PMC13213687; doi:10.1016/j.crmicr.2026.100606)
Supplement: Supplementary file 2 [file mmc2.docx]

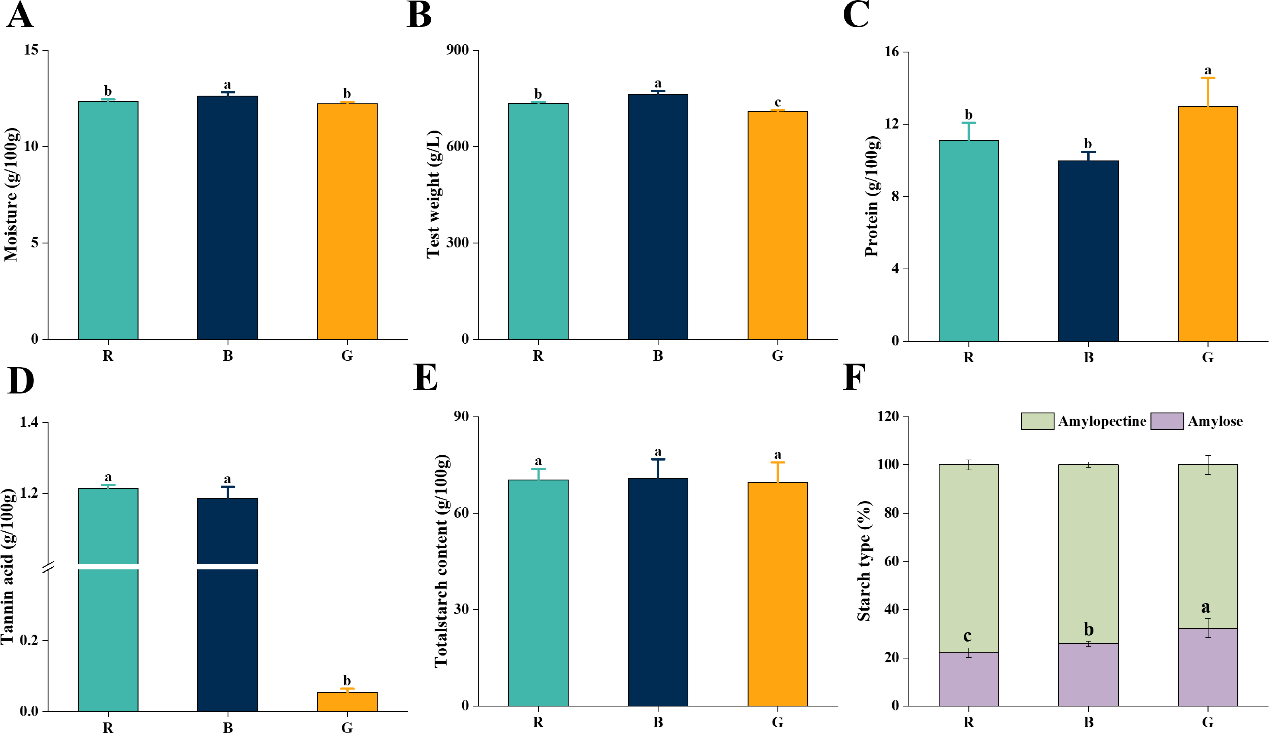


**Figure S1:** The chemical compositions of sorghum. (A) water content. (B) test weight. (C) protein. (D) tannin acid. (E) total starch content. (F) starch type.

R denotes *Hongyingzi* sorghum. B denotes *Hei’e* sorghum. G denotes *Shandong* sorghum.


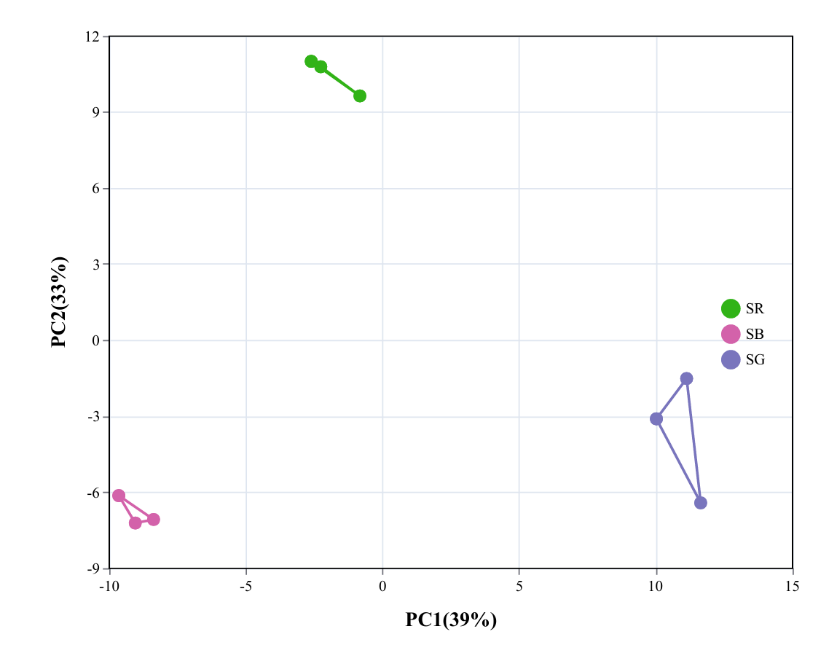


**Figure S2:** Principal component analysis (PCA) based on volatiles in sesame-flavor *Baijiu* (SFB) samples.


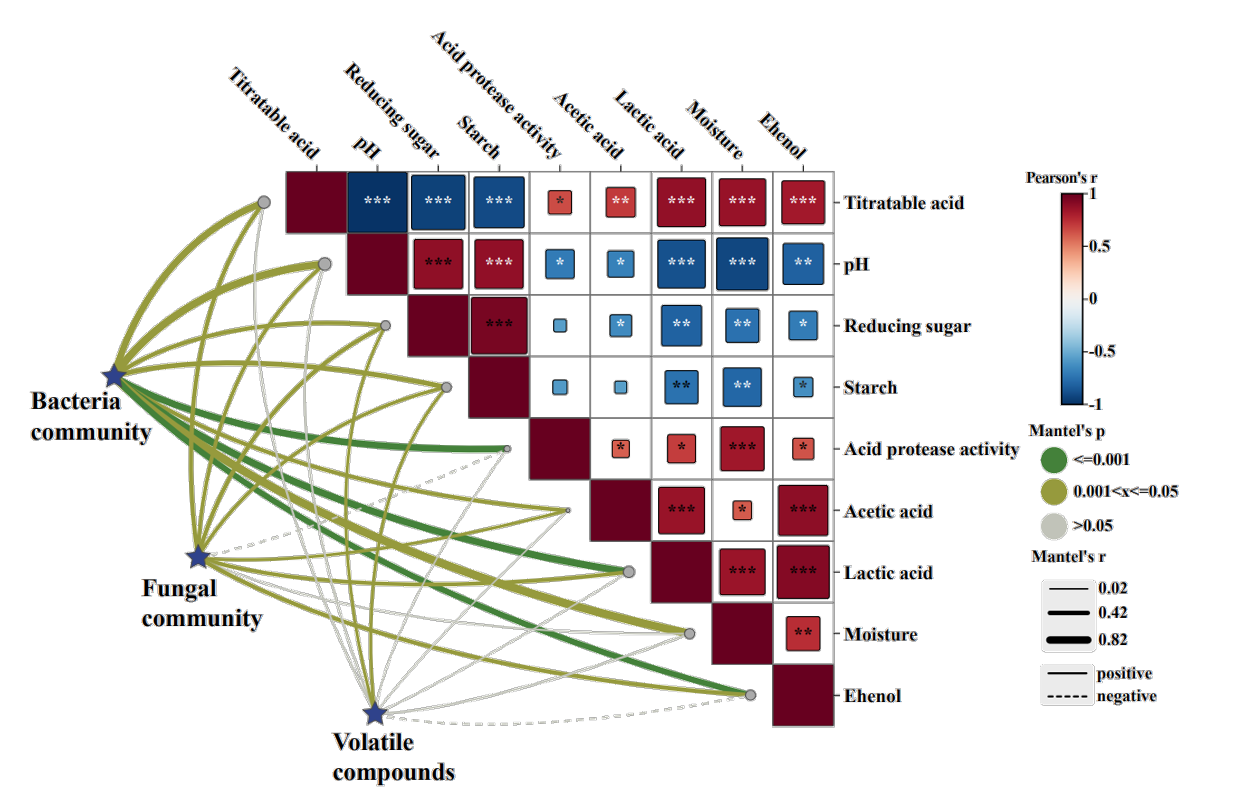


**Figure S3:** Mantel correlation test among physicochemical factors, bacterial/fungal flora and volatile profiles during fermentation.


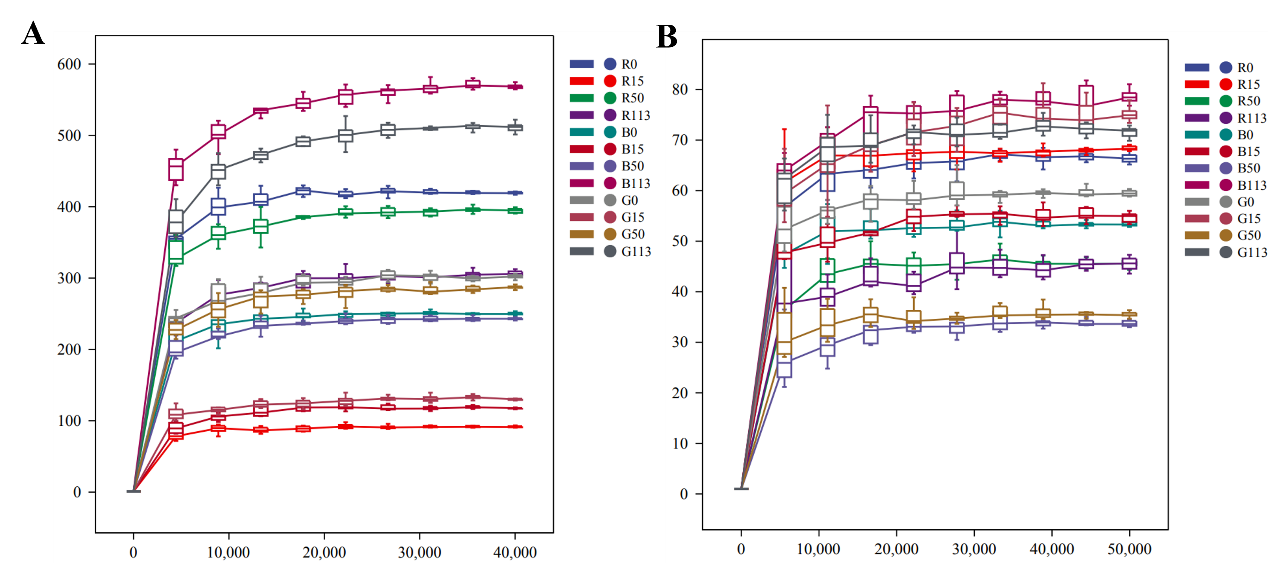


**Figure S4:** Rarefaction curves of sequencing data.


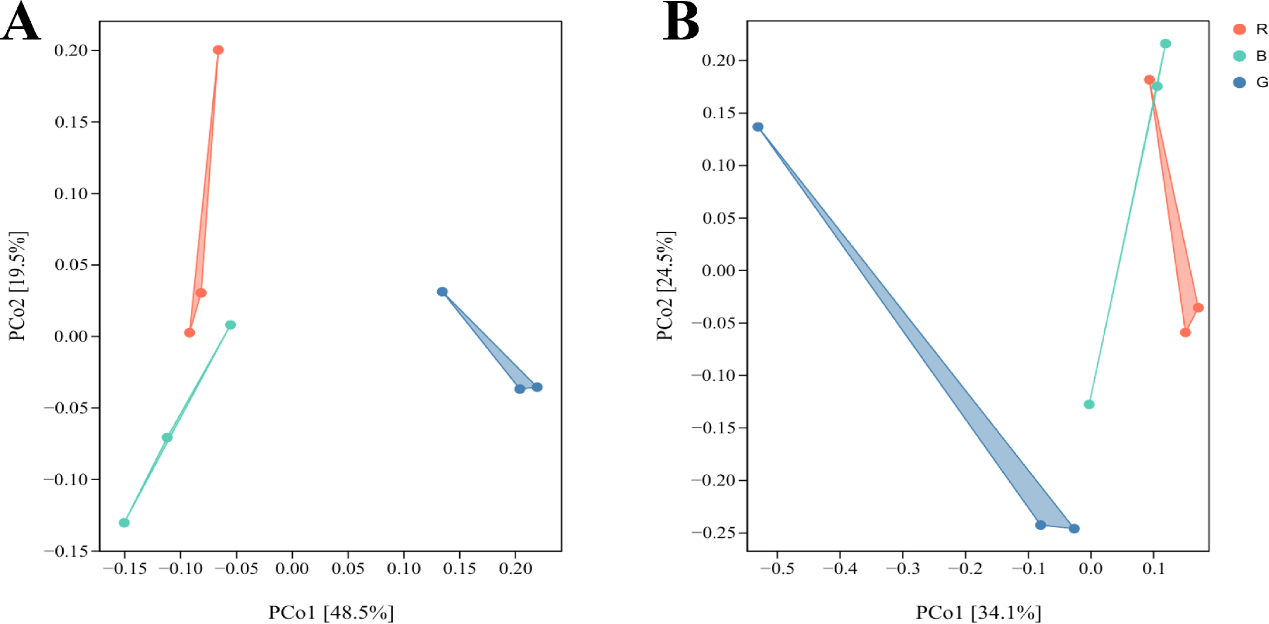


**Figure S5:** Changes in microbial communities β-diversity at the end of fermentation in three varieties of sorghum. (A) Bacterial communities. (B) Fungal communities.

Table S1. Differences in volatile compounds of three SFB analyzed by Random Forests Analysis

| **Name** | **B** | **G** | **R** | **MeanDecreaseAccuracy** | **MeanDecreaseGini** |
| --- | --- | --- | --- | --- | --- |
| 2-Acetyl-5-methylfuran | 2.18803953 | 1.3428499 | 3.0676227 | 3.17959808 | 0.08408889 |
| Pyrazine, 2-ethyl-6-methyl- | 2.65506034 | 2.33008977 | 2.72070143 | 3.18582007 | 0.07635556 |
| Acetic acid, 2-(4-fluorophenyl)-, ethyl ester | 1.66898632 | 1.4156299 | 2.40734225 | 2.36458217 | 0.07445556 |
| 1H-Pyrrole, 1-(2-furanylmethyl)- | 1.63517485 | 1.4156299 | 1.94511995 | 2.14926178 | 0.06848889 |
| 4-Octenoic acid, ethyl ether | 2.65506034 | 1.63517485 | 2.33008977 | 2.9444771 | 0.06276667 |
| Heptanoic acid, ethyl ester | 1.63517485 | 2.00401204 | -0.5774465 | 1.70809204 | 0.05624444 |
| Benzeneacetic acid, ethyl ester | 1.94511995 | 2.14299382 | 1.4156299 | 2.31157098 | 0.05495556 |
| 3,5-Di-tert-butyl-2-hydroxybenzonitrile | 1.66898632 | 2.11529601 | 1.21357078 | 2.35054283 | 0.05385556 |
| Hexanoic acid, ethyl ester | 2.14299382 | 1.63517485 | 1.4156299 | 2.31688103 | 0.05321111 |
| Phenol, 4-chloro-2,6-bis(1,1-dimethyleth | 2.3635973 | 0 | 2.2416792 | 2.30095741 | 0.05155556 |
| Azulene, 1,4-dimethyl-7-(1-methylethyl)- | 3.29743069 | 1.9007911 | 1.63517485 | 3.17387495 | 0.05121111 |
| Butanedioic acid, ethyl 3-methylbutyl | 2.14299382 | 1.3428499 | 0 | 2.11578545 | 0.05052222 |
| Benzonitrile | 1.66898632 | 2.00401204 | 3.17820863 | 3.05855947 | 0.05026667 |
| Furan, 2,2'-methylenebis- | 0 | 1.94511995 | 2.5668229 | 2.5942586 | 0.05022222 |
| Benzene, 1-ethenyl-4-methoxy- | 0 | 1.26592421 | 2.33008977 | 1.95436434 | 0.04966667 |
| Phenol, 4-ethyl-2-methoxy- | -1.6689863 | 2.72070143 | 1.09173949 | 1.72158878 | 0.04942222 |
| Mequinol | 1.94511995 | 2.75603367 | 1.4156299 | 2.81336677 | 0.04873333 |
| 4-Heptenoic acid, ethyl ester, (E)- | 2.40734225 | 1.00050038 | 1.3428499 | 2.30890047 | 0.04845556 |
| Phenylacetic acid, 2-methylbutyl ester | 1.3428499 | 1.94511995 | 1.00050038 | 1.88897926 | 0.04673333 |
| 3,5-Di-tert-butyl-2-hydroxybenzaldehyde | 0 | 2.11529601 | 2.60883281 | 2.55347685 | 0.0458 |
| Octanoic acid, 3-methylbutyl ester | 1.94511995 | 1.00050038 | 2.3635973 | 2.2053434 | 0.04565556 |
| 1-Octanol | 2.14299382 | 2.31558422 | 0 | 2.27263625 | 0.04555556 |
| Isoamyl lactate | 1.4156299 | 1.3428499 | 2.18803953 | 2.13344712 | 0.04531111 |
| Pentanoic acid, 1,1-dimethylpropyl ester | 1.4156299 | 1.3428499 | 1.94511995 | 1.9007911 | 0.04514444 |
| 1,3-Cyclododecadiene, (E,Z)- | 1.63517485 | 1.4156299 | 2.2416792 | 2.2051127 | 0.04505556 |
| Butanedioic acid, ethyl 3-methylbutyl ester | 2.2416792 | 1.00050038 | 1.94511995 | 2.16168851 | 0.04494444 |
| Butanoic acid | 1.66898632 | 2.50396388 | 1.00050038 | 2.45071541 | 0.04465556 |
| 1-Octen-3-ol | 1.3428499 | 1.66898632 | -1.3428499 | 1.13462305 | 0.04332222 |
| Benzene, 1-methoxy-4-methyl- | 1.4156299 | 1.66898632 | -1.0005004 | 1.32303861 | 0.04311111 |
| Phenylacetic acid, propyl ester | 1.73465474 | 0 | 1.66898632 | 1.66678995 | 0.04268889 |
| Benzaldehyde, 3,4-dimethyl- | 2.5668229 | 1.06965637 | 1.4156299 | 2.30575242 | 0.04264444 |
| Acetic acid, phenyl-, isopentyl ester | -1.1346231 | 2.2416792 | 1.4156299 | 1.26271344 | 0.04241111 |
| Hexanoic acid, butyl ester | 1.00050038 | 1.9007911 | 2.72070143 | 2.5762702 | 0.0422 |
| Phenylglyoxylic acid, 2-methylpropyl ester | 1.3428499 | 1.4156299 | 1.9007911 | 1.98379104 | 0.04132222 |
| Oxirane, [[4-(1,1-dimethylethyl)phenoxy] | 1.66898632 | 1.4156299 | 0 | 1.62542249 | 0.04077778 |
| p-Hexylacetophenone | 1.00050038 | 1.00050038 | 1.9007911 | 1.91353979 | 0.04061111 |
| Pentanoic acid, 3-methylbutyl ester | 0 | 1.66898632 | 1.00050038 | 1.32688607 | 0.04058889 |
| Bicyclo[4.2.0]octa-1,3,5-triene, 7-methyl- | 2.2416792 | 1.3428499 | 1.4156299 | 2.09705338 | 0.04025556 |
| Benzenepropanoic acid, ethyl ester | -1.0005004 | 1.3428499 | 1.4156299 | 1.21307462 | 0.03981111 |
| Linalool | 0.44725832 | 2.18803953 | 2.3635973 | 2.40734225 | 0.03957778 |
| Butanoic acid, 3-methyl-, 2-phenylethyl ester | -1.3428499 | 1.89320611 | 1.94511995 | 1.13071015 | 0.0389 |
| Butanoic acid, 2-furanylmethyl ester | 1.00050038 | 0.44725832 | 1.66898632 | 1.66678995 | 0.03872222 |
| Benzene, 1,2-dimethoxy- | 1.00050038 | 1.4156299 | 1.00050038 | 1.4156299 | 0.03818889 |
| Propanoic acid, 2-methyl-, 3-phenylpropyl ester | 1.73465474 | 2.3635973 | 1.4156299 | 2.28908257 | 0.03807778 |
| Diethyl methylsuccinate | 2.60883281 | 0.33335185 | 1.66898632 | 2.31157098 | 0.03781111 |
| Heptane, 2,3-dimethyl- | 1.3428499 | 1.73465474 | 2.5668229 | 2.59669708 | 0.03756667 |
| Naphthalene | 2.33008977 | 1.4156299 | 1.00050038 | 2.11071462 | 0.03753333 |
| Dimethyl phthalate | 0 | 2.3635973 | 1.73465474 | 2.23872261 | 0.03728889 |
| Dodecane, 2,6,11-trimethyl- | 1.4156299 | 1.00050038 | 1.00050038 | 1.25373916 | 0.03688889 |
| Phenol, 4-ethyl- | 1.4156299 | 1.4156299 | -1.0005004 | 1.30096692 | 0.0367 |
| Naphthalene, 1,6-dimethyl-4-(1-methylethyl)- | 1.3428499 | 1.00050038 | 1.94511995 | 1.80292311 | 0.03627778 |
| Octanoic acid | 1.00050038 | 1.3428499 | 2.14299382 | 1.98084135 | 0.03572222 |
| 2-Fluorobenzoic acid, 2,4,6-trichlorophenyl ester | 1.3428499 | 2.14299382 | 1.00050038 | 2.09705338 | 0.03566667 |
| Acetic acid, octyl ester | 1.00050038 | 2.45687145 | 1.3428499 | 2.22853707 | 0.03516667 |
| Thujone | -0.4472583 | 2.18803953 | 2.11529601 | 2.37014512 | 0.03516667 |
| Butanoic acid, 3-methylbutyl ester | -1.0005004 | 1.00050038 | 0.33335185 | 0.47896807 | 0.03453333 |
| Ethanone, 1,2-furanyl- | 0 | 2.45687145 | 2.00401204 | 2.35054283 | 0.0345 |
| Benzoic acid, 2-methylpropyl ester | 0.33335185 | 0 | 1.00050038 | 0.969504 | 0.03436667 |
| 8-Methylnonanoic acid, ethyl ester | 1.00050038 | 1.3428499 | 2.00401204 | 1.95035597 | 0.03367778 |
| 4-Methoxy-3-buten-2-one | 1.4156299 | 2.00401204 | 0 | 1.86266267 | 0.03315556 |
| 2,3-Dimethyl-5-ethylpyrazine | 2.18803953 | 1.3428499 | 1.66898632 | 2.33248926 | 0.03273333 |
| Naphthalene, 2-methyl- | 2.2416792 | 1.4156299 | 0 | 1.82625224 | 0.03262222 |
| Propanoic acid, 2-hydroxy-, 2-methylpropyl ester | 1.4156299 | 1.00050038 | 1.73465474 | 1.72045197 | 0.03258889 |
| Thiophene-2-carboxylic acid ethyl ester | 2.3635973 | 1.4156299 | 1.3428499 | 2.19838265 | 0.03255556 |
| Benzene, 1-(1,5-dimethyl-4-hexenyl)-4-methyl- | 1.66898632 | 1.00050038 | 2.18803953 | 2.17303812 | 0.03238889 |
| N-(1-Cyanocyclopropyl)formamide | 1.3428499 | 1.3428499 | 2.2416792 | 2.37014512 | 0.03237778 |
| 2,6-Octadien-1-ol, 3,7-dimethyl-, (Z)- | 0 | 1.3428499 | 1.73465474 | 1.61374306 | 0.03207778 |
| Butanoic acid, 3-methyl-, 2-furanylmethyl ester | 1.3428499 | 1.00050038 | 1.63517485 | 1.71751347 | 0.03204444 |
| Ethyl 2-hydroxy-3-phenylpropanoate | 0.57744652 | 1.66898632 | -1.0005004 | 0.81126506 | 0.03203333 |
| 1-Heptadecene | 1.00050038 | 1.00050038 | 1.66898632 | 1.60563317 | 0.03151111 |
| β-Phenylethyl butyrate | 1.00050038 | 0 | 1.00050038 | 1.00050038 | 0.03055556 |
| Benzeneacetic acid, 2-methylpropyl ester | 1.9007911 | 2.5668229 | 1.3428499 | 2.52260717 | 0.03054444 |
| Benzaldehyde, 2,5-dimethyl- | 1.4156299 | 1.00050038 | 1.00050038 | 1.38808583 | 0.0303 |
| 4-Cyclopentene-1,3-dione | 0.44725832 | 1.3428499 | 0 | 1.00050038 | 0.02993333 |
| Benzene, 1,3-bis(1,1-dimethylethyl)- | -1.3428499 | 2.2416792 | 1.66898632 | 1.86374954 | 0.02987778 |
| Propanoic acid, 2-methyl-, 2-phenylethyl ester | 1.63517485 | -1.0005004 | 1.3428499 | 1.63517485 | 0.02975556 |
| 3,7,11-Trimethyl-3-hydroxy-6,10-dodecadien-1-yl acetate | 1.00050038 | 2.00401204 | 1.3428499 | 1.94157486 | 0.02952222 |
| Propanoic acid, 2-methyl- | 1.63517485 | 0 | 1.00050038 | 1.70020143 | 0.02933333 |
| Furfural | 2.33008977 | 1.3428499 | 0 | 2.12171053 | 0.02928889 |
| Furfuryl ethyl ether | 1.94511995 | -0.5774465 | 1.60563317 | 1.55504052 | 0.029 |
| 3-Thiopheneacetic acid | 1.3428499 | 1.00050038 | 1.66898632 | 1.63517485 | 0.02887778 |
| Dimethyl trisulfide | 1.00050038 | 1.94511995 | 1.3428499 | 1.94157486 | 0.02887778 |
| Butanoic acid, 3-methyl- | 1.3428499 | 1.3428499 | 2.18803953 | 2.18200253 | 0.02872222 |
| Benzenemethanol, 2-methyl-, acetate | 2.00401204 | 0 | 1.4156299 | 1.9007911 | 0.02854444 |
| 1-Butanol, 3-methyl-, benzoate | 1.4156299 | -1.6689863 | 1.00050038 | 0 | 0.02825556 |
| 1-Dodecanol | 0 | 0 | 1.00050038 | 1.00050038 | 0.02807778 |
| 1-Cyclohexene-1-carboxaldehyde, 2,6,6-trimethyl- | 2.00401204 | 1.73465474 | 1.00050038 | 1.9409757 | 0.02796667 |
| Butanoic acid, 3-methyl-, 3-methylbutyl ester | 1.4156299 | 2.2416792 | 1.3428499 | 2.18180146 | 0.02795556 |
| 1-Decanol | 1.66898632 | -1.2659242 | 0.57744652 | 0.35480977 | 0.02783333 |
| Benzene, (2,2-diethoxyethyl)- | 1.4156299 | 1.00050038 | 0.57744652 | 1.4156299 | 0.02771111 |
| 2-Decanone | 1.4156299 | 1.00050038 | 2.00401204 | 1.91587229 | 0.02753333 |
| 2-Furaldehyde diethyl acetal | 0 | 1.4156299 | 1.3428499 | 1.40137402 | 0.02747778 |
| 1-Naphthalenol, 2-methyl- | 1.4156299 | 2.40734225 | 1.63517485 | 2.34169024 | 0.02742222 |
| 2-Propanamine,N-(1,1-dimethylethyl)-N-hydroxy-2-methyl- | 1.94511995 | 1.00050038 | 1.63517485 | 1.98638874 | 0.02733333 |
| Butanoic acid, ethyl ester | 2.14299382 | 1.00050038 | 1.73465474 | 2.06210138 | 0.0273 |
| Acetic acid, phenylmethyl ester | -1.0005004 | 1.94511995 | 1.4156299 | 1.65730319 | 0.02722222 |
| Benzaldehyde, 2,4,6-trimethyl- | 2.00401204 | 1.3428499 | 1.4156299 | 2.00124634 | 0.0272 |
| Formic acid, heptyl ester | 1.00050038 | 1.66898632 | 1.9007911 | 1.98379104 | 0.02714444 |
| 2-Undecanol | 1.00050038 | 0 | 1.00050038 | 1.00050038 | 0.02696667 |
| Pentanoic acid, 2-hydroxy-4-methyl-, eth | 1.4156299 | 2.2416792 | 1.4156299 | 2.3084031 | 0.02692222 |
| Furfuryl pentanoate | 2.18803953 | 1.73465474 | 1.66898632 | 2.26445945 | 0.02624444 |
| Isopentyl hexanoate | 1.4156299 | 1.94511995 | 1.00050038 | 1.85247534 | 0.02621111 |
| 6-Methyl-6-(5-methylfuran-2-yl)heptan-2-one | 1.4156299 | 0 | 1.00050038 | 1.24614059 | 0.02577778 |
| 8-Nonenoic acid, ethyl ester | 1.00050038 | 1.00050038 | 1.00050038 | 1.21357078 | 0.02552222 |
| Ethanone, 1-(2,3-dihydro-1H-inden-5-yl)- | 0 | 0 | 0 | 0 | 0.02537778 |
| α-Terpineol | -1.0005004 | 0 | 1.00050038 | 0 | 0.02502222 |
| Propanoic acid, 2-methyl-, 2-phenylethyl | 1.63517485 | 0 | 1.4156299 | 1.59674682 | 0.02474444 |
| Pentadecanoic acid, 3-methylbutyl ester | 1.00050038 | 1.3428499 | 1.00050038 | 1.54789239 | 0.02472222 |
| 3-(Methylthio)propanoic acid ethyl ester | 2.2416792 | 1.4156299 | 0 | 1.95035597 | 0.02442222 |
| Benzoic acid, ethyl ester | 0 | 1.00050038 | -1.0005004 | 0 | 0.02427778 |
| Benzaldehyde | 0.44725832 | 0 | -1.4156299 | -0.4685727 | 0.02408889 |
| Pyrazine, trimethyl- | 1.4156299 | 1.00050038 | 1.00050038 | 1.46310196 | 0.0239 |
| 2-Furanpropanoic acid, ethyl ester | 0 | 1.4156299 | 1.3428499 | 1.38808583 | 0.02351111 |
| 5-Methyl-2-phenyl-2-hexenal | 1.4156299 | 1.9007911 | 1.00050038 | 2.1065265 | 0.0234 |
| Butyl lactate | 1.66898632 | 2.40734225 | 1.4156299 | 2.51776572 | 0.02328889 |
| Benzene, 1,3-bis(1,1-dimethylethyl)-5-methyl- | 0 | 1.3428499 | 1.00050038 | 1.3428499 | 0.022 |
| Benzene, (1,1-dimethyl-2-butynyl)- | 1.3428499 | 2.3635973 | 1.00050038 | 2.21735718 | 0.02176667 |
| Pyrrolidine-2,5-dione, 1-(2-nitro-3-pyridyl)- | 1.00050038 | 1.00050038 | 0 | 1.00050038 | 0.02151111 |
| 2-Nonen-1-ol, (E)- | 1.00050038 | 1.4156299 | 1.00050038 | 1.40137402 | 0.02131111 |
| Methoxyacetic acid, 3-methylbutyl-ester | 0 | 0 | 1.00050038 | 1.00050038 | 0.02107778 |
| 3-Octen-2-one | 0 | 2.40734225 | 1.89320611 | 2.28024504 | 0.02094444 |
| 7(1H)-Pteridinone | 2.45687145 | 1.89320611 | 1.3428499 | 2.43993123 | 0.02076667 |
| Formic acid, octyl ester | 0 | 1.3428499 | 1.4156299 | 1.4156299 | 0.0206 |
| 1H-Imidazole-4-carboxylic acid, 2,5-dimethyl-, ethyl ester | 2.3635973 | 1.00050038 | 1.3428499 | 2.33645407 | 0.02022222 |
| Diethyl malonate | 0 | 1.00050038 | 0 | 1.00050038 | 0.02014444 |
| Benzaldehyde, 4-methyl- | 1.00050038 | 1.00050038 | 1.3428499 | 1.40137402 | 0.01965556 |
| Butanoic acid, 2-hydroxy-, ethyl ester | 1.4156299 | 0 | 1.66898632 | 1.52931047 | 0.01955556 |
| Ethyl 9-hexadecenoate | -1.0005004 | 1.4156299 | 1.26592421 | 1.12868752 | 0.01926667 |
| Butanoic acid, 2-methyl-, 2-methylbutyl ester | 0 | 1.73465474 | 1.00050038 | 1.59201047 | 0.01905556 |
| 4-Decenoic acid, ethyl ester, (Z)- | 1.73465474 | 1.4156299 | 1.00050038 | 1.70836987 | 0.01901111 |
| 1-Hexanol, 2-ethyl- | -0.5774465 | -1.0005004 | 1.00050038 | -0.0957831 | 0.01895556 |
| 1, 1, 5-Trimethyl-1, 2-dihydronaphthalen | -1.4156299 | 1.4156299 | -0.5774465 | -0.4752446 | 0.01886667 |
| Pentanoic acid, 4-oxo-, ethyl ester | 1.00050038 | 1.00050038 | 1.00050038 | 1.38808583 | 0.01864444 |
| Ethanone, 2-(formyloxy)-1-phenyl- | 1.00050038 | 1.4156299 | 0 | 1.3428499 | 0.01856667 |
| 2,4-Di-tert-butylphenol | -1.3428499 | 0.44725832 | 1.00050038 | -0.3180154 | 0.01848889 |
| Undecanoic acid, ethyl ester | 1.73465474 | 0.33335185 | 0.27736077 | 1.24196721 | 0.01837778 |
| Nonanoic acid, ethyl ester | 0 | -1.0005004 | 0 | -1.0005004 | 0.01818889 |
| trans-2-Undecen-1-ol | 1.9007911 | 1.3428499 | 1.3428499 | 2.11414855 | 0.01817778 |
| 2-Cyclopentene-1-carboxylic acid, 1,2,3-trimethyl-, ethyl ester, (.+-.)- | 1.3428499 | 1.3428499 | 1.89320611 | 2.11414855 | 0.01807778 |
| 2-Furanbutanoic acid, γ-oxo- | 1.4156299 | 0 | 1.4156299 | 1.40695434 | 0.01801111 |
| Benzofuran | 0 | 1.4156299 | 1.4156299 | 1.4156299 | 0.01784444 |
| 2(3H)-Benzofuranone, 3-methyl- | 1.4156299 | 1.00050038 | 1.00050038 | 1.30096692 | 0.01771111 |
| Furan, 2,2'-[oxybis(methylene)]bis- | 1.00050038 | 1.4156299 | 1.00050038 | 1.3428499 | 0.01756667 |
| n-Caprylic acid isobutyl ester | 0 | 0 | 1.00050038 | 1.00050038 | 0.01728889 |
| Heptanoic acid, 3-methylbutyl ester | 1.4156299 | 1.73465474 | 1.00050038 | 1.6973029 | 0.01691111 |
| Hexanoic acid, anhydride | 1.00050038 | 0 | 0 | 1.00050038 | 0.01668889 |
| β-Chloro-para-fluoropropiophenone | 1.9007911 | 1.4156299 | 1.00050038 | 1.91353979 | 0.01661111 |
| Butanoic acid, butyl ester | 1.3428499 | 1.4156299 | 1.00050038 | 1.7258454 | 0.01624444 |
| Butanedioic acid, diethyl ester | 0 | 0 | 0 | 0 | 0.01622222 |
| Benzeneacetic acid, butyl ester | 1.9007911 | 1.00050038 | 1.3428499 | 2.19616737 | 0.01586667 |
| Benzyl alcohol | 2.18803953 | 1.3428499 | 1.4156299 | 2.11583128 | 0.01581111 |
| Diethyl suberate | 1.73465474 | 0 | 1.3428499 | 1.52931047 | 0.01472222 |
| Phenylethyl Alcohol | 0 | 0 | 0 | 0 | 0.01451111 |
| 2-Buten-1-one, 1-(2,6,6-trimethyl-1,3-cyclohexadien-1-yl)-, (E)- | 0 | 0 | 0 | 0 | 0.01447778 |
| 1-Heptanol | 1.3428499 | 1.66898632 | 0 | 1.63046761 | 0.01423333 |
| p-Heptylaniline | 1.4156299 | 1.73465474 | 1.00050038 | 1.66678995 | 0.01411111 |
| 2-Naphthalenethiol | 0 | 0 | -1.0005004 | -1.0005004 | 0.01406667 |
| 2-Furanmethanol | 1.63517485 | 1.00050038 | 0 | 1.73465474 | 0.01391111 |
| 6-Heptenoic acid, ethyl ester | 0 | 1.00050038 | 1.00050038 | 1.00050038 | 0.01374444 |
| 2-Nonanone | 0 | 0 | -1.0005004 | -1.0005004 | 0.01323333 |
| Butanoic acid, 2-methyl-, 2-phenylethyl ester | 1.00050038 | 1.3428499 | 1.00050038 | 1.40137402 | 0.01307778 |
| 7-Octenoic acid, ethyl ester | 1.00050038 | 0 | 1.00050038 | 1.40137402 | 0.01305556 |
| 2-Furancarboxylic acid, ethyl ester | 0 | 0 | -1.0005004 | -1.0005004 | 0.01203333 |
| 3-Octanol | 0 | 1.3428499 | 1.00050038 | 1.40137402 | 0.01198889 |
| Pentane, 2,2-dimethyl- | 1.3428499 | 1.00050038 | 0 | 1.37930743 | 0.0116 |
| Terpinen-4-ol | 0 | 0 | 0 | 0 | 0.01141111 |
| Undecanoic acid, 2-methyl- | 1.00050038 | -1.0005004 | 0 | 0 | 0.01134444 |
| Acetic acid, 2-phenylethyl ester | 0 | 0 | 1.00050038 | 1.00050038 | 0.0113 |
| 1-Butanol, 3-methyl- | -1.0005004 | -1.3428499 | -1.0005004 | -1.3428499 | 0.01093333 |
| 1-Nonanol | -1.0005004 | -0.4472583 | 0 | -0.585306 | 0.01093333 |
| Decanoic acid, ethyl ester | -1.0005004 | -1.0005004 | 0 | -1.0005004 | 0.01088889 |
| Benzaldehyde, 4-propyl- | 0 | -1.0005004 | -1.0005004 | -1.0005004 | 0.01057778 |
| Acetic acid, nonyl ester | -1.0005004 | 1.00050038 | 0 | 0.200004 | 0.01023333 |
| 2-Pentadecanone | 0 | 0 | 0 | 0 | 0.01021111 |
| 2-Pyrazoline, 1-isopropyl-3,4-dimethyl- | 0 | -1.0005004 | -1.4156299 | -1.3880858 | 0.01017778 |
| 3-Nonenoic acid, ethyl ester | -1.0005004 | 0 | 0 | -1.0005004 | 0.01017778 |
| Tetradecanoic acid, ethyl ester | 0 | 0 | -1.0005004 | -1.0005004 | 0.00998889 |
| 2-Furanmethanol, acetate | 1.00050038 | 0 | 0 | 1.00050038 | 0.00941111 |
| Ethyl trans-4-decenoate | 0 | -1.0005004 | 0 | -1.0005004 | 0.00933333 |
| Octanoic acid, ethyl ester | 0 | 1.00050038 | 1.00050038 | 1.00050038 | 0.00932222 |
| 1-Hexanol | 0 | 0 | -1.0005004 | -1.0005004 | 0.00861111 |
| Ethanone, 2-cyclopentyl-1-(1H-imidazol-4 | 1.00050038 | 1.00050038 | 0 | 1.00050038 | 0.00773333 |
| Ethyl trans-2-decenoate | 0 | -1.0005004 | -1.0005004 | -1.0005004 | 0.007 |
| Thymol | -1.3428499 | 0 | 0 | -1.3428499 | 0.0069 |
| Pentanedioic acid, diethyl ester | 0 | -1.0005004 | 0 | -1.0005004 | 0.00673333 |
| Styrene | 1.00050038 | 1.00050038 | -1.4156299 | 0 | 0.00672222 |
| Benzene, 1,2,4,5-tetramethyl- | 1.00050038 | 0 | 0 | 1.00050038 | 0.00533333 |
| Diethyl azelate | 0 | 0 | 0 | 0 | 0.00523333 |
| Dodecanoic acid, ethyl ester | -1.0005004 | -1.0005004 | 0 | -1.0005004 | 0.00487778 |
| Furan, 3-phenyl- | 0 | 0 | 0 | 0 | 0.0048 |
| Hexadecanoic acid, ethyl ester | 0 | 0 | 0 | 0 | 0.00471111 |
| Pentadecanoic acid, ethyl ester | 1.00050038 | 0 | 1.00050038 | 1.00050038 | 0.0044 |
| Propanamide, N-ethyl-N-(3-methylphenyl)-2-chloro- | 0 | 0 | 0 | 0 | 0.004 |
| Propanoic acid, 2-hydroxy-, ethyl ester, (L)- | -1.3428499 | 0 | -1.0005004 | -1.2885209 | 0.0039 |
| 2-Tridecanone | 1.00050038 | 0 | 0 | 1.00050038 | 0.0015 |
| 2-Undecanone | 0 | 0 | 0 | 0 | 0.0015 |

R denotes *Hongyingzi* sorghum. B denotes *Hei’e* sorghum. G denotes *Shandong* sorghum.

Table S2 Pairs of primers developed in this study

| Amplified Fragment |  | | Sequence (5’-3’) |
| --- | --- | --- | --- |
| 16S rRNA | Forward | 799F (AACMGGATTAGATACCCKG) | |
|  | Reverse | 1193R (ACGTCATCCCCACCTTCC) | |
| ITS1(b) | Forward | ITS1F (CTTGGTCATTTAGAGGAAGTAA) | |
|  | Reverse | ITS2 (GCTGCGTTCTTCATCGATGC) | |
