# Supplementary material for: Multi-omics analysis-based insights into the microbial community composition and flavor development potentiality of different varieties of sorghum (Sorghum bicolor L. Moench) fermented into Sesame flavor Baijiu
Source: Curr Res Microb Sci. 2026 May 15;10:100606. doi: 10.1016/j.crmicr.2026.100606 (PMC13213687; doi:10.1016/j.crmicr.2026.100606)
Supplement: Supplementary file 3 [file mmc3.docx]

Table S1. Differences in volatile compounds of three SFB analyzed by Random Forests Analysis

| **Name** | **B** | **G** | **R** | **MeanDecreaseAccuracy** | **MeanDecreaseGini** |
| --- | --- | --- | --- | --- | --- |
| 2-Acetyl-5-methylfuran | 2.18803953 | 1.3428499 | 3.0676227 | 3.17959808 | 0.08408889 |
| Pyrazine, 2-ethyl-6-methyl- | 2.65506034 | 2.33008977 | 2.72070143 | 3.18582007 | 0.07635556 |
| Acetic acid, 2-(4-fluorophenyl)-, ethyl ester | 1.66898632 | 1.4156299 | 2.40734225 | 2.36458217 | 0.07445556 |
| 1H-Pyrrole, 1-(2-furanylmethyl)- | 1.63517485 | 1.4156299 | 1.94511995 | 2.14926178 | 0.06848889 |
| 4-Octenoic acid, ethyl ether | 2.65506034 | 1.63517485 | 2.33008977 | 2.9444771 | 0.06276667 |
| Heptanoic acid, ethyl ester | 1.63517485 | 2.00401204 | -0.5774465 | 1.70809204 | 0.05624444 |
| Benzeneacetic acid, ethyl ester | 1.94511995 | 2.14299382 | 1.4156299 | 2.31157098 | 0.05495556 |
| 3,5-Di-tert-butyl-2-hydroxybenzonitrile | 1.66898632 | 2.11529601 | 1.21357078 | 2.35054283 | 0.05385556 |
| Hexanoic acid, ethyl ester | 2.14299382 | 1.63517485 | 1.4156299 | 2.31688103 | 0.05321111 |
| Phenol, 4-chloro-2,6-bis(1,1-dimethyleth | 2.3635973 | 0 | 2.2416792 | 2.30095741 | 0.05155556 |
| Azulene, 1,4-dimethyl-7-(1-methylethyl)- | 3.29743069 | 1.9007911 | 1.63517485 | 3.17387495 | 0.05121111 |
| Butanedioic acid, ethyl 3-methylbutyl | 2.14299382 | 1.3428499 | 0 | 2.11578545 | 0.05052222 |
| Benzonitrile | 1.66898632 | 2.00401204 | 3.17820863 | 3.05855947 | 0.05026667 |
| Furan, 2,2'-methylenebis- | 0 | 1.94511995 | 2.5668229 | 2.5942586 | 0.05022222 |
| Benzene, 1-ethenyl-4-methoxy- | 0 | 1.26592421 | 2.33008977 | 1.95436434 | 0.04966667 |
| Phenol, 4-ethyl-2-methoxy- | -1.6689863 | 2.72070143 | 1.09173949 | 1.72158878 | 0.04942222 |
| Mequinol | 1.94511995 | 2.75603367 | 1.4156299 | 2.81336677 | 0.04873333 |
| 4-Heptenoic acid, ethyl ester, (E)- | 2.40734225 | 1.00050038 | 1.3428499 | 2.30890047 | 0.04845556 |
| Phenylacetic acid, 2-methylbutyl ester | 1.3428499 | 1.94511995 | 1.00050038 | 1.88897926 | 0.04673333 |
| 3,5-Di-tert-butyl-2-hydroxybenzaldehyde | 0 | 2.11529601 | 2.60883281 | 2.55347685 | 0.0458 |
| Octanoic acid, 3-methylbutyl ester | 1.94511995 | 1.00050038 | 2.3635973 | 2.2053434 | 0.04565556 |
| 1-Octanol | 2.14299382 | 2.31558422 | 0 | 2.27263625 | 0.04555556 |
| Isoamyl lactate | 1.4156299 | 1.3428499 | 2.18803953 | 2.13344712 | 0.04531111 |
| Pentanoic acid, 1,1-dimethylpropyl ester | 1.4156299 | 1.3428499 | 1.94511995 | 1.9007911 | 0.04514444 |
| 1,3-Cyclododecadiene, (E,Z)- | 1.63517485 | 1.4156299 | 2.2416792 | 2.2051127 | 0.04505556 |
| Butanedioic acid, ethyl 3-methylbutyl ester | 2.2416792 | 1.00050038 | 1.94511995 | 2.16168851 | 0.04494444 |
| Butanoic acid | 1.66898632 | 2.50396388 | 1.00050038 | 2.45071541 | 0.04465556 |
| 1-Octen-3-ol | 1.3428499 | 1.66898632 | -1.3428499 | 1.13462305 | 0.04332222 |
| Benzene, 1-methoxy-4-methyl- | 1.4156299 | 1.66898632 | -1.0005004 | 1.32303861 | 0.04311111 |
| Phenylacetic acid, propyl ester | 1.73465474 | 0 | 1.66898632 | 1.66678995 | 0.04268889 |
| Benzaldehyde, 3,4-dimethyl- | 2.5668229 | 1.06965637 | 1.4156299 | 2.30575242 | 0.04264444 |
| Acetic acid, phenyl-, isopentyl ester | -1.1346231 | 2.2416792 | 1.4156299 | 1.26271344 | 0.04241111 |
| Hexanoic acid, butyl ester | 1.00050038 | 1.9007911 | 2.72070143 | 2.5762702 | 0.0422 |
| Phenylglyoxylic acid, 2-methylpropyl ester | 1.3428499 | 1.4156299 | 1.9007911 | 1.98379104 | 0.04132222 |
| Oxirane, [[4-(1,1-dimethylethyl)phenoxy] | 1.66898632 | 1.4156299 | 0 | 1.62542249 | 0.04077778 |
| p-Hexylacetophenone | 1.00050038 | 1.00050038 | 1.9007911 | 1.91353979 | 0.04061111 |
| Pentanoic acid, 3-methylbutyl ester | 0 | 1.66898632 | 1.00050038 | 1.32688607 | 0.04058889 |
| Bicyclo[4.2.0]octa-1,3,5-triene, 7-methyl- | 2.2416792 | 1.3428499 | 1.4156299 | 2.09705338 | 0.04025556 |
| Benzenepropanoic acid, ethyl ester | -1.0005004 | 1.3428499 | 1.4156299 | 1.21307462 | 0.03981111 |
| Linalool | 0.44725832 | 2.18803953 | 2.3635973 | 2.40734225 | 0.03957778 |
| Butanoic acid, 3-methyl-, 2-phenylethyl ester | -1.3428499 | 1.89320611 | 1.94511995 | 1.13071015 | 0.0389 |
| Butanoic acid, 2-furanylmethyl ester | 1.00050038 | 0.44725832 | 1.66898632 | 1.66678995 | 0.03872222 |
| Benzene, 1,2-dimethoxy- | 1.00050038 | 1.4156299 | 1.00050038 | 1.4156299 | 0.03818889 |
| Propanoic acid, 2-methyl-, 3-phenylpropyl ester | 1.73465474 | 2.3635973 | 1.4156299 | 2.28908257 | 0.03807778 |
| Diethyl methylsuccinate | 2.60883281 | 0.33335185 | 1.66898632 | 2.31157098 | 0.03781111 |
| Heptane, 2,3-dimethyl- | 1.3428499 | 1.73465474 | 2.5668229 | 2.59669708 | 0.03756667 |
| Naphthalene | 2.33008977 | 1.4156299 | 1.00050038 | 2.11071462 | 0.03753333 |
| Dimethyl phthalate | 0 | 2.3635973 | 1.73465474 | 2.23872261 | 0.03728889 |
| Dodecane, 2,6,11-trimethyl- | 1.4156299 | 1.00050038 | 1.00050038 | 1.25373916 | 0.03688889 |
| Phenol, 4-ethyl- | 1.4156299 | 1.4156299 | -1.0005004 | 1.30096692 | 0.0367 |
| Naphthalene, 1,6-dimethyl-4-(1-methylethyl)- | 1.3428499 | 1.00050038 | 1.94511995 | 1.80292311 | 0.03627778 |
| Octanoic acid | 1.00050038 | 1.3428499 | 2.14299382 | 1.98084135 | 0.03572222 |
| 2-Fluorobenzoic acid, 2,4,6-trichlorophenyl ester | 1.3428499 | 2.14299382 | 1.00050038 | 2.09705338 | 0.03566667 |
| Acetic acid, octyl ester | 1.00050038 | 2.45687145 | 1.3428499 | 2.22853707 | 0.03516667 |
| Thujone | -0.4472583 | 2.18803953 | 2.11529601 | 2.37014512 | 0.03516667 |
| Butanoic acid, 3-methylbutyl ester | -1.0005004 | 1.00050038 | 0.33335185 | 0.47896807 | 0.03453333 |
| Ethanone, 1,2-furanyl- | 0 | 2.45687145 | 2.00401204 | 2.35054283 | 0.0345 |
| Benzoic acid, 2-methylpropyl ester | 0.33335185 | 0 | 1.00050038 | 0.969504 | 0.03436667 |
| 8-Methylnonanoic acid, ethyl ester | 1.00050038 | 1.3428499 | 2.00401204 | 1.95035597 | 0.03367778 |
| 4-Methoxy-3-buten-2-one | 1.4156299 | 2.00401204 | 0 | 1.86266267 | 0.03315556 |
| 2,3-Dimethyl-5-ethylpyrazine | 2.18803953 | 1.3428499 | 1.66898632 | 2.33248926 | 0.03273333 |
| Naphthalene, 2-methyl- | 2.2416792 | 1.4156299 | 0 | 1.82625224 | 0.03262222 |
| Propanoic acid, 2-hydroxy-, 2-methylpropyl ester | 1.4156299 | 1.00050038 | 1.73465474 | 1.72045197 | 0.03258889 |
| Thiophene-2-carboxylic acid ethyl ester | 2.3635973 | 1.4156299 | 1.3428499 | 2.19838265 | 0.03255556 |
| Benzene, 1-(1,5-dimethyl-4-hexenyl)-4-methyl- | 1.66898632 | 1.00050038 | 2.18803953 | 2.17303812 | 0.03238889 |
| N-(1-Cyanocyclopropyl)formamide | 1.3428499 | 1.3428499 | 2.2416792 | 2.37014512 | 0.03237778 |
| 2,6-Octadien-1-ol, 3,7-dimethyl-, (Z)- | 0 | 1.3428499 | 1.73465474 | 1.61374306 | 0.03207778 |
| Butanoic acid, 3-methyl-, 2-furanylmethyl ester | 1.3428499 | 1.00050038 | 1.63517485 | 1.71751347 | 0.03204444 |
| Ethyl 2-hydroxy-3-phenylpropanoate | 0.57744652 | 1.66898632 | -1.0005004 | 0.81126506 | 0.03203333 |
| 1-Heptadecene | 1.00050038 | 1.00050038 | 1.66898632 | 1.60563317 | 0.03151111 |
| β-Phenylethyl butyrate | 1.00050038 | 0 | 1.00050038 | 1.00050038 | 0.03055556 |
| Benzeneacetic acid, 2-methylpropyl ester | 1.9007911 | 2.5668229 | 1.3428499 | 2.52260717 | 0.03054444 |
| Benzaldehyde, 2,5-dimethyl- | 1.4156299 | 1.00050038 | 1.00050038 | 1.38808583 | 0.0303 |
| 4-Cyclopentene-1,3-dione | 0.44725832 | 1.3428499 | 0 | 1.00050038 | 0.02993333 |
| Benzene, 1,3-bis(1,1-dimethylethyl)- | -1.3428499 | 2.2416792 | 1.66898632 | 1.86374954 | 0.02987778 |
| Propanoic acid, 2-methyl-, 2-phenylethyl ester | 1.63517485 | -1.0005004 | 1.3428499 | 1.63517485 | 0.02975556 |
| 3,7,11-Trimethyl-3-hydroxy-6,10-dodecadien-1-yl acetate | 1.00050038 | 2.00401204 | 1.3428499 | 1.94157486 | 0.02952222 |
| Propanoic acid, 2-methyl- | 1.63517485 | 0 | 1.00050038 | 1.70020143 | 0.02933333 |
| Furfural | 2.33008977 | 1.3428499 | 0 | 2.12171053 | 0.02928889 |
| Furfuryl ethyl ether | 1.94511995 | -0.5774465 | 1.60563317 | 1.55504052 | 0.029 |
| 3-Thiopheneacetic acid | 1.3428499 | 1.00050038 | 1.66898632 | 1.63517485 | 0.02887778 |
| Dimethyl trisulfide | 1.00050038 | 1.94511995 | 1.3428499 | 1.94157486 | 0.02887778 |
| Butanoic acid, 3-methyl- | 1.3428499 | 1.3428499 | 2.18803953 | 2.18200253 | 0.02872222 |
| Benzenemethanol, 2-methyl-, acetate | 2.00401204 | 0 | 1.4156299 | 1.9007911 | 0.02854444 |
| 1-Butanol, 3-methyl-, benzoate | 1.4156299 | -1.6689863 | 1.00050038 | 0 | 0.02825556 |
| 1-Dodecanol | 0 | 0 | 1.00050038 | 1.00050038 | 0.02807778 |
| 1-Cyclohexene-1-carboxaldehyde, 2,6,6-trimethyl- | 2.00401204 | 1.73465474 | 1.00050038 | 1.9409757 | 0.02796667 |
| Butanoic acid, 3-methyl-, 3-methylbutyl ester | 1.4156299 | 2.2416792 | 1.3428499 | 2.18180146 | 0.02795556 |
| 1-Decanol | 1.66898632 | -1.2659242 | 0.57744652 | 0.35480977 | 0.02783333 |
| Benzene, (2,2-diethoxyethyl)- | 1.4156299 | 1.00050038 | 0.57744652 | 1.4156299 | 0.02771111 |
| 2-Decanone | 1.4156299 | 1.00050038 | 2.00401204 | 1.91587229 | 0.02753333 |
| 2-Furaldehyde diethyl acetal | 0 | 1.4156299 | 1.3428499 | 1.40137402 | 0.02747778 |
| 1-Naphthalenol, 2-methyl- | 1.4156299 | 2.40734225 | 1.63517485 | 2.34169024 | 0.02742222 |
| 2-Propanamine,N-(1,1-dimethylethyl)-N-hydroxy-2-methyl- | 1.94511995 | 1.00050038 | 1.63517485 | 1.98638874 | 0.02733333 |
| Butanoic acid, ethyl ester | 2.14299382 | 1.00050038 | 1.73465474 | 2.06210138 | 0.0273 |
| Acetic acid, phenylmethyl ester | -1.0005004 | 1.94511995 | 1.4156299 | 1.65730319 | 0.02722222 |
| Benzaldehyde, 2,4,6-trimethyl- | 2.00401204 | 1.3428499 | 1.4156299 | 2.00124634 | 0.0272 |
| Formic acid, heptyl ester | 1.00050038 | 1.66898632 | 1.9007911 | 1.98379104 | 0.02714444 |
| 2-Undecanol | 1.00050038 | 0 | 1.00050038 | 1.00050038 | 0.02696667 |
| Pentanoic acid, 2-hydroxy-4-methyl-, eth | 1.4156299 | 2.2416792 | 1.4156299 | 2.3084031 | 0.02692222 |
| Furfuryl pentanoate | 2.18803953 | 1.73465474 | 1.66898632 | 2.26445945 | 0.02624444 |
| Isopentyl hexanoate | 1.4156299 | 1.94511995 | 1.00050038 | 1.85247534 | 0.02621111 |
| 6-Methyl-6-(5-methylfuran-2-yl)heptan-2-one | 1.4156299 | 0 | 1.00050038 | 1.24614059 | 0.02577778 |
| 8-Nonenoic acid, ethyl ester | 1.00050038 | 1.00050038 | 1.00050038 | 1.21357078 | 0.02552222 |
| Ethanone, 1-(2,3-dihydro-1H-inden-5-yl)- | 0 | 0 | 0 | 0 | 0.02537778 |
| α-Terpineol | -1.0005004 | 0 | 1.00050038 | 0 | 0.02502222 |
| Propanoic acid, 2-methyl-, 2-phenylethyl | 1.63517485 | 0 | 1.4156299 | 1.59674682 | 0.02474444 |
| Pentadecanoic acid, 3-methylbutyl ester | 1.00050038 | 1.3428499 | 1.00050038 | 1.54789239 | 0.02472222 |
| 3-(Methylthio)propanoic acid ethyl ester | 2.2416792 | 1.4156299 | 0 | 1.95035597 | 0.02442222 |
| Benzoic acid, ethyl ester | 0 | 1.00050038 | -1.0005004 | 0 | 0.02427778 |
| Benzaldehyde | 0.44725832 | 0 | -1.4156299 | -0.4685727 | 0.02408889 |
| Pyrazine, trimethyl- | 1.4156299 | 1.00050038 | 1.00050038 | 1.46310196 | 0.0239 |
| 2-Furanpropanoic acid, ethyl ester | 0 | 1.4156299 | 1.3428499 | 1.38808583 | 0.02351111 |
| 5-Methyl-2-phenyl-2-hexenal | 1.4156299 | 1.9007911 | 1.00050038 | 2.1065265 | 0.0234 |
| Butyl lactate | 1.66898632 | 2.40734225 | 1.4156299 | 2.51776572 | 0.02328889 |
| Benzene, 1,3-bis(1,1-dimethylethyl)-5-methyl- | 0 | 1.3428499 | 1.00050038 | 1.3428499 | 0.022 |
| Benzene, (1,1-dimethyl-2-butynyl)- | 1.3428499 | 2.3635973 | 1.00050038 | 2.21735718 | 0.02176667 |
| Pyrrolidine-2,5-dione, 1-(2-nitro-3-pyridyl)- | 1.00050038 | 1.00050038 | 0 | 1.00050038 | 0.02151111 |
| 2-Nonen-1-ol, (E)- | 1.00050038 | 1.4156299 | 1.00050038 | 1.40137402 | 0.02131111 |
| Methoxyacetic acid, 3-methylbutyl-ester | 0 | 0 | 1.00050038 | 1.00050038 | 0.02107778 |
| 3-Octen-2-one | 0 | 2.40734225 | 1.89320611 | 2.28024504 | 0.02094444 |
| 7(1H)-Pteridinone | 2.45687145 | 1.89320611 | 1.3428499 | 2.43993123 | 0.02076667 |
| Formic acid, octyl ester | 0 | 1.3428499 | 1.4156299 | 1.4156299 | 0.0206 |
| 1H-Imidazole-4-carboxylic acid, 2,5-dimethyl-, ethyl ester | 2.3635973 | 1.00050038 | 1.3428499 | 2.33645407 | 0.02022222 |
| Diethyl malonate | 0 | 1.00050038 | 0 | 1.00050038 | 0.02014444 |
| Benzaldehyde, 4-methyl- | 1.00050038 | 1.00050038 | 1.3428499 | 1.40137402 | 0.01965556 |
| Butanoic acid, 2-hydroxy-, ethyl ester | 1.4156299 | 0 | 1.66898632 | 1.52931047 | 0.01955556 |
| Ethyl 9-hexadecenoate | -1.0005004 | 1.4156299 | 1.26592421 | 1.12868752 | 0.01926667 |
| Butanoic acid, 2-methyl-, 2-methylbutyl ester | 0 | 1.73465474 | 1.00050038 | 1.59201047 | 0.01905556 |
| 4-Decenoic acid, ethyl ester, (Z)- | 1.73465474 | 1.4156299 | 1.00050038 | 1.70836987 | 0.01901111 |
| 1-Hexanol, 2-ethyl- | -0.5774465 | -1.0005004 | 1.00050038 | -0.0957831 | 0.01895556 |
| 1, 1, 5-Trimethyl-1, 2-dihydronaphthalen | -1.4156299 | 1.4156299 | -0.5774465 | -0.4752446 | 0.01886667 |
| Pentanoic acid, 4-oxo-, ethyl ester | 1.00050038 | 1.00050038 | 1.00050038 | 1.38808583 | 0.01864444 |
| Ethanone, 2-(formyloxy)-1-phenyl- | 1.00050038 | 1.4156299 | 0 | 1.3428499 | 0.01856667 |
| 2,4-Di-tert-butylphenol | -1.3428499 | 0.44725832 | 1.00050038 | -0.3180154 | 0.01848889 |
| Undecanoic acid, ethyl ester | 1.73465474 | 0.33335185 | 0.27736077 | 1.24196721 | 0.01837778 |
| Nonanoic acid, ethyl ester | 0 | -1.0005004 | 0 | -1.0005004 | 0.01818889 |
| trans-2-Undecen-1-ol | 1.9007911 | 1.3428499 | 1.3428499 | 2.11414855 | 0.01817778 |
| 2-Cyclopentene-1-carboxylic acid, 1,2,3-trimethyl-, ethyl ester, (.+-.)- | 1.3428499 | 1.3428499 | 1.89320611 | 2.11414855 | 0.01807778 |
| 2-Furanbutanoic acid, γ-oxo- | 1.4156299 | 0 | 1.4156299 | 1.40695434 | 0.01801111 |
| Benzofuran | 0 | 1.4156299 | 1.4156299 | 1.4156299 | 0.01784444 |
| 2(3H)-Benzofuranone, 3-methyl- | 1.4156299 | 1.00050038 | 1.00050038 | 1.30096692 | 0.01771111 |
| Furan, 2,2'-[oxybis(methylene)]bis- | 1.00050038 | 1.4156299 | 1.00050038 | 1.3428499 | 0.01756667 |
| n-Caprylic acid isobutyl ester | 0 | 0 | 1.00050038 | 1.00050038 | 0.01728889 |
| Heptanoic acid, 3-methylbutyl ester | 1.4156299 | 1.73465474 | 1.00050038 | 1.6973029 | 0.01691111 |
| Hexanoic acid, anhydride | 1.00050038 | 0 | 0 | 1.00050038 | 0.01668889 |
| β-Chloro-para-fluoropropiophenone | 1.9007911 | 1.4156299 | 1.00050038 | 1.91353979 | 0.01661111 |
| Butanoic acid, butyl ester | 1.3428499 | 1.4156299 | 1.00050038 | 1.7258454 | 0.01624444 |
| Butanedioic acid, diethyl ester | 0 | 0 | 0 | 0 | 0.01622222 |
| Benzeneacetic acid, butyl ester | 1.9007911 | 1.00050038 | 1.3428499 | 2.19616737 | 0.01586667 |
| Benzyl alcohol | 2.18803953 | 1.3428499 | 1.4156299 | 2.11583128 | 0.01581111 |
| Diethyl suberate | 1.73465474 | 0 | 1.3428499 | 1.52931047 | 0.01472222 |
| Phenylethyl Alcohol | 0 | 0 | 0 | 0 | 0.01451111 |
| 2-Buten-1-one, 1-(2,6,6-trimethyl-1,3-cyclohexadien-1-yl)-, (E)- | 0 | 0 | 0 | 0 | 0.01447778 |
| 1-Heptanol | 1.3428499 | 1.66898632 | 0 | 1.63046761 | 0.01423333 |
| p-Heptylaniline | 1.4156299 | 1.73465474 | 1.00050038 | 1.66678995 | 0.01411111 |
| 2-Naphthalenethiol | 0 | 0 | -1.0005004 | -1.0005004 | 0.01406667 |
| 2-Furanmethanol | 1.63517485 | 1.00050038 | 0 | 1.73465474 | 0.01391111 |
| 6-Heptenoic acid, ethyl ester | 0 | 1.00050038 | 1.00050038 | 1.00050038 | 0.01374444 |
| 2-Nonanone | 0 | 0 | -1.0005004 | -1.0005004 | 0.01323333 |
| Butanoic acid, 2-methyl-, 2-phenylethyl ester | 1.00050038 | 1.3428499 | 1.00050038 | 1.40137402 | 0.01307778 |
| 7-Octenoic acid, ethyl ester | 1.00050038 | 0 | 1.00050038 | 1.40137402 | 0.01305556 |
| 2-Furancarboxylic acid, ethyl ester | 0 | 0 | -1.0005004 | -1.0005004 | 0.01203333 |
| 3-Octanol | 0 | 1.3428499 | 1.00050038 | 1.40137402 | 0.01198889 |
| Pentane, 2,2-dimethyl- | 1.3428499 | 1.00050038 | 0 | 1.37930743 | 0.0116 |
| Terpinen-4-ol | 0 | 0 | 0 | 0 | 0.01141111 |
| Undecanoic acid, 2-methyl- | 1.00050038 | -1.0005004 | 0 | 0 | 0.01134444 |
| Acetic acid, 2-phenylethyl ester | 0 | 0 | 1.00050038 | 1.00050038 | 0.0113 |
| 1-Butanol, 3-methyl- | -1.0005004 | -1.3428499 | -1.0005004 | -1.3428499 | 0.01093333 |
| 1-Nonanol | -1.0005004 | -0.4472583 | 0 | -0.585306 | 0.01093333 |
| Decanoic acid, ethyl ester | -1.0005004 | -1.0005004 | 0 | -1.0005004 | 0.01088889 |
| Benzaldehyde, 4-propyl- | 0 | -1.0005004 | -1.0005004 | -1.0005004 | 0.01057778 |
| Acetic acid, nonyl ester | -1.0005004 | 1.00050038 | 0 | 0.200004 | 0.01023333 |
| 2-Pentadecanone | 0 | 0 | 0 | 0 | 0.01021111 |
| 2-Pyrazoline, 1-isopropyl-3,4-dimethyl- | 0 | -1.0005004 | -1.4156299 | -1.3880858 | 0.01017778 |
| 3-Nonenoic acid, ethyl ester | -1.0005004 | 0 | 0 | -1.0005004 | 0.01017778 |
| Tetradecanoic acid, ethyl ester | 0 | 0 | -1.0005004 | -1.0005004 | 0.00998889 |
| 2-Furanmethanol, acetate | 1.00050038 | 0 | 0 | 1.00050038 | 0.00941111 |
| Ethyl trans-4-decenoate | 0 | -1.0005004 | 0 | -1.0005004 | 0.00933333 |
| Octanoic acid, ethyl ester | 0 | 1.00050038 | 1.00050038 | 1.00050038 | 0.00932222 |
| 1-Hexanol | 0 | 0 | -1.0005004 | -1.0005004 | 0.00861111 |
| Ethanone, 2-cyclopentyl-1-(1H-imidazol-4 | 1.00050038 | 1.00050038 | 0 | 1.00050038 | 0.00773333 |
| Ethyl trans-2-decenoate | 0 | -1.0005004 | -1.0005004 | -1.0005004 | 0.007 |
| Thymol | -1.3428499 | 0 | 0 | -1.3428499 | 0.0069 |
| Pentanedioic acid, diethyl ester | 0 | -1.0005004 | 0 | -1.0005004 | 0.00673333 |
| Styrene | 1.00050038 | 1.00050038 | -1.4156299 | 0 | 0.00672222 |
| Benzene, 1,2,4,5-tetramethyl- | 1.00050038 | 0 | 0 | 1.00050038 | 0.00533333 |
| Diethyl azelate | 0 | 0 | 0 | 0 | 0.00523333 |
| Dodecanoic acid, ethyl ester | -1.0005004 | -1.0005004 | 0 | -1.0005004 | 0.00487778 |
| Furan, 3-phenyl- | 0 | 0 | 0 | 0 | 0.0048 |
| Hexadecanoic acid, ethyl ester | 0 | 0 | 0 | 0 | 0.00471111 |
| Pentadecanoic acid, ethyl ester | 1.00050038 | 0 | 1.00050038 | 1.00050038 | 0.0044 |
| Propanamide, N-ethyl-N-(3-methylphenyl)-2-chloro- | 0 | 0 | 0 | 0 | 0.004 |
| Propanoic acid, 2-hydroxy-, ethyl ester, (L)- | -1.3428499 | 0 | -1.0005004 | -1.2885209 | 0.0039 |
| 2-Tridecanone | 1.00050038 | 0 | 0 | 1.00050038 | 0.0015 |
| 2-Undecanone | 0 | 0 | 0 | 0 | 0.0015 |

R denotes *Hongyingzi* sorghum. B denotes *Hei’e* sorghum. G denotes *Shandong* sorghum.

Table S2 Pairs of primers developed in this study

| Amplified Fragment |  | | Sequence (5’-3’) |
| --- | --- | --- | --- |
| 16S rRNA | Forward | 799F (AACMGGATTAGATACCCKG) | |
|  | Reverse | 1193R (ACGTCATCCCCACCTTCC) | |
| ITS1(b) | Forward | ITS1F (CTTGGTCATTTAGAGGAAGTAA) | |
|  | Reverse | ITS2 (GCTGCGTTCTTCATCGATGC) | |
